# YTHDF2-mediated regulations bifurcate BHPF-induced programmed cell deaths

**DOI:** 10.1093/nsr/nwad227

**Published:** 2023-08-28

**Authors:** Jiebo Lin, Guankai Zhan, Jinfeng Liu, Yasen Maimaitiyiming, Zhiping Deng, Baohua Li, Kunhui Su, Jiafeng Chen, Siqi Sun, Wanlin Zheng, Xianghui Yu, Feng He, Xiaodong Cheng, Lingfang Wang, Bin Shen, Ziqin Yao, Xinquan Yang, Jian Zhang, Wentao He, Hengyu Wu, Hua Naranmandura, Kao-Jung Chang, Junxia Min, Jun Ma, Mikael Björklund, Peng-Fei Xu, Fudi Wang, Chih-Hung Hsu

**Affiliations:** Women's Hospital, The Fourth Affiliated Hospital, and Department of Environmental Medicine, Zhejiang University School of Medicine, Hangzhou 310006; Institute of Genetics, International School of Medicine, Zhejiang University, Yiwu 322000; Women's Hospital, The Fourth Affiliated Hospital, and Department of Environmental Medicine, Zhejiang University School of Medicine, Hangzhou 310006; Institute of Genetics, International School of Medicine, Zhejiang University, Yiwu 322000; Women's Hospital, The Fourth Affiliated Hospital, and Department of Environmental Medicine, Zhejiang University School of Medicine, Hangzhou 310006; Institute of Genetics, International School of Medicine, Zhejiang University, Yiwu 322000; The Second Affiliated Hospital, School of Public Health, State Key Laboratory of Experimental Hematology, Zhejiang University School of Medicine, Hangzhou 310058; Women's Hospital, The Fourth Affiliated Hospital, and Department of Environmental Medicine, Zhejiang University School of Medicine, Hangzhou 310006; Institute of Genetics, International School of Medicine, Zhejiang University, Yiwu 322000; Department of Hematology of First Affiliated Hospital, and Department of Public Health, Zhejiang University School of Medicine, Hangzhou 310000; State Key Laboratory for Managing Biotic and Chemical Threats to the Quality and Safety of Agro-products, Institute of Virology and Biotechnology, Zhejiang Academy of Agricultural Sciences, Hangzhou 310000; Department of Obstetrics, Women's Hospital, School of Medicine, Zhejiang University, Hangzhou 310000; Women's Hospital, The Fourth Affiliated Hospital, and Department of Environmental Medicine, Zhejiang University School of Medicine, Hangzhou 310006; Institute of Genetics, International School of Medicine, Zhejiang University, Yiwu 322000; Women's Hospital, The Fourth Affiliated Hospital, and Department of Environmental Medicine, Zhejiang University School of Medicine, Hangzhou 310006; Institute of Genetics, International School of Medicine, Zhejiang University, Yiwu 322000; Women's Hospital, The Fourth Affiliated Hospital, and Department of Environmental Medicine, Zhejiang University School of Medicine, Hangzhou 310006; Institute of Genetics, International School of Medicine, Zhejiang University, Yiwu 322000; Women's Hospital, and Institute of Genetics, Zhejiang University School of Medicine, Hangzhou 310000; Zhejiang Provincial Key Lab of Genetic and Developmental Disorders, Hangzhou 310058; Women's Hospital, and Institute of Genetics, Zhejiang University School of Medicine, Hangzhou 310000; Zhejiang Provincial Key Lab of Genetic and Developmental Disorders, Hangzhou 310058; Institute of Genetics, International School of Medicine, Zhejiang University, Yiwu 322000; Women's Hospital, and Institute of Genetics, Zhejiang University School of Medicine, Hangzhou 310000; Zhejiang Provincial Key Lab of Genetic and Developmental Disorders, Hangzhou 310058; Zhejiang Provincial Key Laboratory of Precision Diagnosis and Therapy for Major Gynecological Diseases, Women's Hospital, Zhejiang University School of Medicine, Hangzhou 310000; Women's Hospital, The Fourth Affiliated Hospital, and Department of Environmental Medicine, Zhejiang University School of Medicine, Hangzhou 310006; Institute of Genetics, International School of Medicine, Zhejiang University, Yiwu 322000; Zhejiang Provincial Key Laboratory of Precision Diagnosis and Therapy for Major Gynecological Diseases, Women's Hospital, Zhejiang University School of Medicine, Hangzhou 310000; State Key Laboratory of Reproductive Medicine, Center for Global Health, Gusu School, Women's Hospital of Nanjing Medical University, Nanjing Maternity and Child Health Care Hospital, Nanjing Medical University, Nanjing 211166; The Second Affiliated Hospital, School of Public Health, State Key Laboratory of Experimental Hematology, Zhejiang University School of Medicine, Hangzhou 310058; The First Affiliated Hospital, Basic Medical Sciences, School of Public Health, School of Pharmaceutical Science, Hengyang Medical School, University of South China, Hengyang 421001; The Second Affiliated Hospital, School of Public Health, State Key Laboratory of Experimental Hematology, Zhejiang University School of Medicine, Hangzhou 310058; The First Affiliated Hospital, Basic Medical Sciences, School of Public Health, School of Pharmaceutical Science, Hengyang Medical School, University of South China, Hengyang 421001; Women's Hospital, The Fourth Affiliated Hospital, and Department of Environmental Medicine, Zhejiang University School of Medicine, Hangzhou 310006; Institute of Genetics, International School of Medicine, Zhejiang University, Yiwu 322000; Women's Hospital, The Fourth Affiliated Hospital, and Department of Environmental Medicine, Zhejiang University School of Medicine, Hangzhou 310006; Institute of Genetics, International School of Medicine, Zhejiang University, Yiwu 322000; Women's Hospital, The Fourth Affiliated Hospital, and Department of Environmental Medicine, Zhejiang University School of Medicine, Hangzhou 310006; Institute of Genetics, International School of Medicine, Zhejiang University, Yiwu 322000; Department of Hematology of First Affiliated Hospital, and Department of Public Health, Zhejiang University School of Medicine, Hangzhou 310000; Department of Medical Research, Taipei Veterans General Hospital, Taipei 11217; The First Affiliated Hospital, Institute of Translational Medicine, Cancer Center, Zhejiang University School of Medicine, Hangzhou 310058; Institute of Genetics, International School of Medicine, Zhejiang University, Yiwu 322000; Women's Hospital, and Institute of Genetics, Zhejiang University School of Medicine, Hangzhou 310000; Zhejiang Provincial Key Lab of Genetic and Developmental Disorders, Hangzhou 310058; Zhejiang University-University of Edinburgh (ZJU-UoE) Institute, Haining 314400; University of Edinburgh Medical School, Biomedical Sciences, College of Medicine & Veterinary Medicine, University of Edinburgh, Edinburgh, EH8 9JZ; Institute of Genetics, International School of Medicine, Zhejiang University, Yiwu 322000; Women's Hospital, and Institute of Genetics, Zhejiang University School of Medicine, Hangzhou 310000; The Second Affiliated Hospital, School of Public Health, State Key Laboratory of Experimental Hematology, Zhejiang University School of Medicine, Hangzhou 310058; The First Affiliated Hospital, Basic Medical Sciences, School of Public Health, School of Pharmaceutical Science, Hengyang Medical School, University of South China, Hengyang 421001; Women's Hospital, The Fourth Affiliated Hospital, and Department of Environmental Medicine, Zhejiang University School of Medicine, Hangzhou 310006; Institute of Genetics, International School of Medicine, Zhejiang University, Yiwu 322000

**Keywords:** RNA m^6^A modification, programmed cell deaths, bifurcation role of YTHDF2, environmental stress and m^6^A regulation, fluorene-9-bisphenol (BHPF)

## Abstract

N^6^-methyladenosine (m^6^A) is a critical regulator in the fate of RNA, but whether and how m^6^A executes its functions in different tissues remains largely obscure. Here we report downregulation of a crucial m^6^A reader, YTHDF2, leading to tissue-specific programmed cell deaths (PCDs) upon fluorene-9-bisphenol (BHPF) exposure. Currently, Bisphenol A (BPA) substitutes are widely used in plastic manufacturing. Interrogating eight common BPA substitutes, we detected BHPF in 14% serum samples of pregnant participants. In a zebrafish model, BHPF caused tissue-specific PCDs triggering cardiac and vascular defects. Mechanistically, BHPF-mediated downregulation of YTHDF2 reduced YTHDF2-facilitated translation of m^6^A-*gch1* for cardiomyocyte ferroptosis, and decreased YTHDF2-mediated m^6^A-*sting1* decay for caudal vein plexus (CVP) apoptosis. The two distinct YTHDF2-mediated m^6^A regulations and context-dependent co-expression patterns of *gch1/ythdf2* and *tnfrsf1a/ythdf2* contributed to YTHDF2-mediated tissue-specific PCDs, uncovering a new layer of PCD regulation. Since BHPF/YTHDF2-medaited PCD defects were also observed in mammals, BHPF exposure represents a potential health threat.

## INTRODUCTION

The extensive application of plastics is a growing public health concern [1–6]. Bisphenol A (BPA), as a major plastic material additive, has been widely applied in the synthesis of polymer materials, many of which are commonly used in food containers, such as milk and beverage bottles, water cups and food packaging [7,8]. Due to its environmental and biological toxicity, especially estrogen toxicity during fetal development, BPA has been restricted by many governments for use in the production of food containers, especially feeding bottles for milk or water. To manufacture ‘BPA-free’ food containers, several BPA substitutes have been introduced to replace BPA. Therefore, evaluation of the effects of these BPA substitutes during early fetal development is urgently needed.

RNA modification is one of the common mechanisms by which environmental stimuli influence cellular functions [9–14], among which N^6^-methyladenosine (m^6^A) extensively occurs on multiple RNA species and is the most prevalent internal modification associated with eukaryotic messenger RNA (mRNA). m^6^A methylation is a reversible and dynamic process modulated by three categories of m^6^A regulatory proteins, including methyltransferases/writers, demethylases/erasers, and binding proteins/readers [15–18]. In fact, the fate of m^6^A-modifided RNA is closely regulated by its readers. Here, we demonstrate that one of the common BPA substitutes, BHPF, could downregulate a crucial m^6^A reader YTHDF2 (YT521-B homology domain family member 2) to influence translation and stability of its downstream RNA targets, respectively, leading to tissue-specific programmed cell deaths (PCDs) and defects.

## RESULTS

### BHPF induces cardiovascular defects in animal models

**Figure 1.**
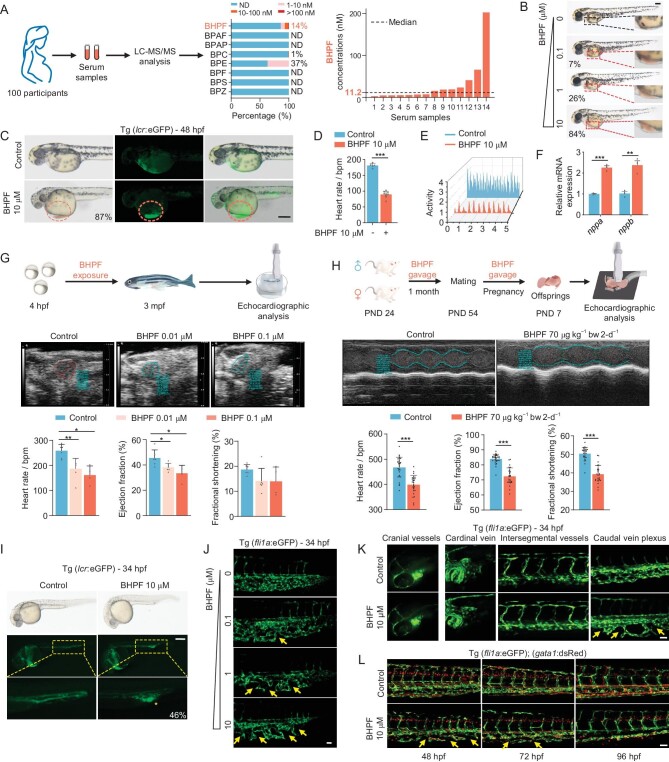
BHPF is detectable in pregnant people and displays cardiac and vascular toxicity in zebrafish and mice. A, Detection rates and concentration ranges of eight BPA substitutes in serum samples from 100 pregnant participants (left panel). Concentration of BHPF in serum samples (right panel). ND refers to not detected. B, Morphological changes of zebrafish (48 hpf) with concentration gradient exposure (0.1–10 μM) to BHPF. The pericardial condensation is indicated by red dashed boxes with percentage; *n* = 25–28 per group. C, Bright field and fluorescence microscopy of BHPF-exposed Tg(*lcr*: eGFP) zebrafish. The red dashed circles indicate pericardial condensation with percentage; *n* = 15–20 per group. D and E, Effects of BHPF (10 μM) on heart rate (D) and heart activity (E) of embryos. Heart rate as beats per minute (bpm) and heart activity recordings (electrocardiogram) were analyzed by DanioScope; *n* = 5–9 per group. F, Effects of BHPF (10 μM) exposure on mRNA expression of *nppa* and *nppb* as detected by RT-qPCR in whole embryos. G, Schematic diagram of echocardiographic analysis for BHPF-exposed zebrafish (top panel). Representative echocardiograms (middle panel), summary of heart rate (as bpm), cardiac ejection fraction (EF) and fractional shortening (FS) in control and BHPF exposed (0.01–0.1 μM for 3 months) zebrafish (bottom panel); *n* = 4–7 per group. H, Diagram showing the BHPF exposure and echocardiography analysis in mice (top panel). Representative echocardiograms (middle panel), summary of heart rate (as bpm), cardiac ejection fraction (EF) and fractional shortening (FS) in the offspring of control and BHPF-exposed mice (7.3 nM BHPF in the serum of pregnant mice) (bottom panel); *n* = 26–29 per group. I, Effects of BHPF (10 μM) on distribution of erythroid in zebrafish (34 hpf). The yellow dashed boxes and asterisk indicate blood accumulation with percentage; *n* = 28–30 per group. J, Confocal observation of CVP in Tg(*fli1a*: eGFP) zebrafish (34 hpf) with concentration gradient exposure (0.1–10 μM) to BHPF. The yellow arrows indicate CVP defects. K, Effects of BHPF (10 μM) on vein development in different regions of zebrafish (34 hpf). The yellow arrows indicate CVP defects. L, Effects of BHPF (10 μM) on vein development in different developmental stages of zebrafish (48–96 hpf). The yellow arrows indicate CVP defects. Zebrafish embryos were treated with BHPF from 4 hpf to the indicated time. Scale bar, 300 μm (B, C, I) or 20 μm (J–L). Data are mean ± s.d. Student's t test, *represents *P* < 0.05, **represents *P* < 0.01, *** represents *P* < 0.001.

We systematically interrogated 8 common BPA substitutes and found that three of them, namely BHPF (CAS NO. 3236-71-3, fluorene-9-bisphenol), BPC (Bisphenol C) and BPE (Bisphenol E), were detectable in the serum of the study participants (100 pregnant people) in varying concentrations and detection rates (Fig. 1A). Their existence in the serum of pregnant participants raised concerns regarding the potential toxicity during fetal development. Thus, we investigated whether these BPA substitutes have adverse effects during fetal development in a zebrafish model. Upon BHPF exposure, apparent pericardial congestion was observed, the detection rate of which increased in a concentration-dependent manner, while the other BPA substitutes did not display such a phenotype (Fig. 1B and Supplementary Fig. 1A). These results suggested that BHPF could induce heart defects during fetal development. Due to the significant detection rate (14%) among pregnant participants and apparent cardiac defects (Fig. 1A, B), we focused on BHPF for further investigation.

To clarify the functional cardiac defects, we exposed the 4 hpf (hours post fertilization) Tg(*lcr*: eGFP) (*lcr*, an erythroid marker gene) transgenic zebrafish embryos to BHPF and observed apparent heart blood stagnation, downregulation of heart rate, and reduction of heart activity (Fig. 1C–E), without affecting the formation of erythroid and myeloid cells (Supplementary Fig. 1B). This suggested that cardiac but not hematopoietic toxicity of BHPF is responsible for the heart defects. Consistently, molecular evidence corroborated the cardiotoxicity of BHPF, as the mRNA levels of heart failure markers *nppa* and *nppb* were increased upon BHPF exposure (Fig. 1F).

The median concentration of the detected BHPF in the serum of pregnant participants was 11.2 nM (Fig. 1A). In the zebrafish model, after acute exposure to 0.1, 1, and 10 μM of BHPF for 24 hours, measured levels of the BHPF were 46, 135, and 427 nM in the fish trunk (Supplementary Fig. 1C). Notably, 0.1 μM (46 nM in the zebrafish body) acute BHPF exposure was able to induce appreciable defects in part of the exposed zebrafish population (Fig. 1B). Moreover, chronic exposure (from 4 hpf to 3 months) to a lower concentration (0.01 μM exposure, 8.6 nM in zebrafish trunks) of BHPF led to reduction of the heart rate, ejection fraction (EF), and fractional shortening (FS) (Fig. 1G and Supplementary Fig. 1D). Furthermore, such BHPF-induced heart defects were also observed in the offspring from BHPF-gavage female mice (70 μg kg^−1^ bw 2-d^−1^ BHPF from postnatal day (PND) 24 for 1 month to pregnancy and birth to PND 7) (7.3 nM BHPF in the serum of pregnant mice) (Fig. 1H). This suggested that the BHPF levels in the blood of pregnant people (median: 11.2 nM) might induce cardiotoxicity.

On the other hand, when we examined BHPF exposed Tg(*lcr*: eGFP) transgenic zebrafish embryos, a specific blood accumulation in the tail region was noticed at around 34 hpf, which precedes the observation of heart defects at 48 hpf (Fig. 1I). Since the formations of erythroid and myeloid cells were not affected upon BHPF exposure (Supplementary Fig. 1B), we further used Tg(*fli1a*: eGFP) (*fli1a*, a vascular endothelial marker gene) transgenic zebrafish to investigate this phenotype and found that only BHPF but not the other examined BPA substitutes caused caudal vein plexus (CVP) defects in a concentration-dependent manner (Fig. 1J and Supplementary Fig. 2A, B). To clearly define the degree of CVP defects, we have calculated loop numbers (asterisks, loops represent network space formed by normal growth extension of initial vessels) and total CVP area (yellow dotted line outlined area) from the confocal images of Tg(*fli1a*: eGFP) transgenic zebrafish. (See online supplementary material for a color version of this figure.) The total CVP area and loop numbers are used to monitor CVP growth and density of branching in CVP. Then these calculated results were used to quantify and define the severity of CVP defect (Supplementary Fig. 2B). Remarkably, BHPF only caused obvious defects in angiogenesis and vascular network formation in CVP but not in other regions (Fig. 1K). Further investigation in Tg(*fli1a*: eGFP); (*gata1*: dsRed) (*gata1:* an erythroid marker gene) transgenic zebrafish showed that BHPF-induced loss of bloodstream around the CVP region persisted at 96 hpf (Fig. 1L), indicating that rather than leading to developmental delay of global vascular networks in zebrafish, BHPF exposure resulted in specific defects of CVP. Collectively, as a common BPA substitute, BHPF is frequently detected in the serum of the investigated pregnant participants (Fig. 1A) and displays apparent cardiac and vascular toxicity to induce heart failure and zebrafish CVP defects (Supplementary Fig. 2C).

### BHPF induces cardiac ferroptosis

**Figure 2.**
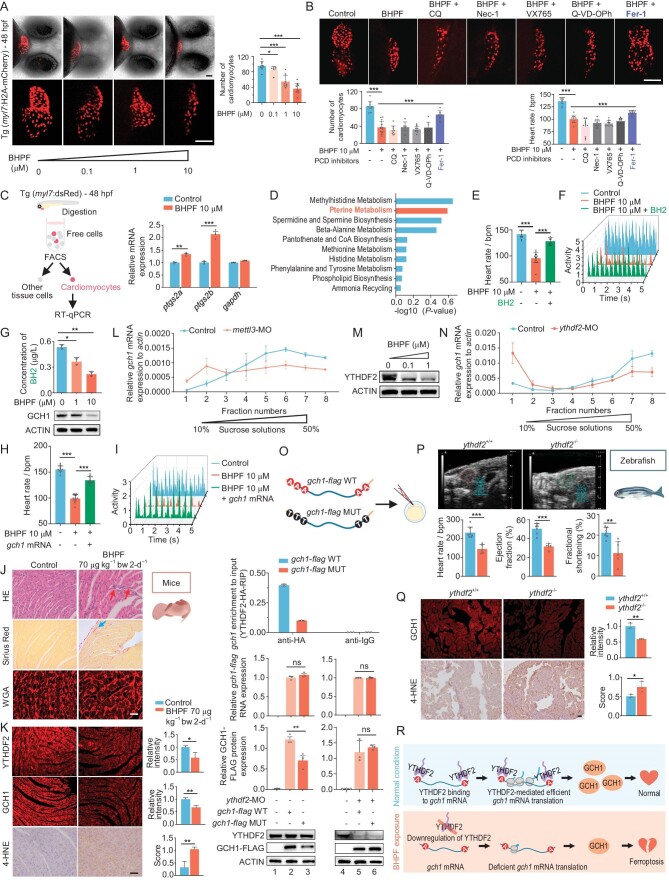
BHPF induces cardiac ferroptosis through reduction of YTHDF2-facilitated translation of m^6^A-*gch1*. A, Changes in the number of zebrafish cardiomyocytes following concentration gradient exposure (0.1–10 μM) to BHPF. The images (left panel) were quantified as a histogram (right panel); *n* = 6–9 per group. B, Rescue effects of different PCD inhibitors on BHPF-induced reduction of heart rate (bpm) and number of cardiomyocytes. Chloroquine (CQ) is an autophagy inhibitor; Necrostatin-1 (Nec-1) is a necroptosis inhibitor; VX765 (Belnacasan) is a pyroptosis inhibitor; Q-VD-OPh is an apoptosis inhibitor; Ferrostatin-1 (Fer-1) is a ferroptosis inhibitor. All inhibitors were co-treated with BHPF from 4 hpf to 48 hpf; *n* = 6–13 per group. C, Effects of BHPF exposure on mRNA expression of ferroptosis markers *ptgs2a* and *ptgs2b* in FACS sorted zebrafish embryonic cardiomyocytes. D, Pathway enrichment analysis by SMPDB (small molecule pathway database) based on metabolomic profiling. E and F, Effects of exogenous BH2 supplementation on heart rate (bpm refers to beats per minute) (E) and heart activity (F) of BHPF exposed zebrafish; *n* = 12–17 per group. G, Effects of BHPF exposure on BH2 concentration and GCH1 protein levels. BH2 concentration was detected by LC-MS/MS. H and I, Rescue effects of exogenous *gch1* mRNA on heart rate (H) and heart activity (I); *n* = 10–12 per group. J, Representative images of cardiac sections stained with Hematoxylin and eosin (HE), Sirius red or Wheat germ agglutinin (WGA) in hearts obtained from the offspring of the control and BHPF-exposed mice. Red arrows indicate inflammatory cells and blue arrows indicate collagen fibrils. K, Images of immunofluorescence for YTHDF2 and GCH1 staining, and immunohistochemistry for 4-HNE in the offspring of the control and BHPF-exposed mice with quantification. L, Measurement of *gch1* mRNA translation profile in *mettl3* morphants by polysome profiling. M, Effects of BHPF exposure on YTHDF2 protein levels. N, Measurement of *gch1* mRNA translation profile in *ythdf2* morphants by polysome profiling. O, Top panel shows the affinity between YTHDF2 and *gch1*-flag mRNA (wild type and m^6^A modification sites mutant). Bottom panels show the measurement of *gch1* mRNA and protein levels in *gch1*-flag (WT and MUT) mRNA microinjected control embryos and *ythdf2* morphants. P, Representative echocardiograms, summary of heart rate, cardiac ejection fraction (EF) and fractional shortening (FS) in wild-type (*ythdf2*^+/+^) and *ythdf2* mutant (*ythdf2*^−/−^) zebrafish; *n* = 5–7 per group. Q, Images of immunofluorescence for GCH1 staining and immunohistochemistry for 4-HNE in wild-type (*ythdf2*^+/+^) and *ythdf2* mutant (*ythdf2*^−/−^) zebrafish with quantification. R, Schematic diagram shows BHPF-mediated downregulation of YTHDF2 stimulating ferroptosis-induced cardiac injury by reduction of YTHDF2-facilitated *gch1* translation. Molecular biology experiment materials are zebrafish embryonic cardiomyocytes sorted by FACS (C) or whole embryos (D, G, L–O). Zebrafish embryos were collected at 48 hpf (A–I, L–M) or 10 hpf (O). Scale bar, 50 μm (A–B) or 20 μm (J–K, Q). Data are mean ± s.d. Student's t test, ns represents *P* > 0.05, * represents *P* < 0.05, ** represents *P* < 0.01, *** represents *P* < 0.001.

To investigate the mechanism by which BHPF induces heart failure during embryonic development, cardiomyocyte marker labeled transgenic zebrafish strains were used. We conducted three-dimensional reconstructions of confocal stacks in BHPF-exposed Tg(*myl7*: H2A-mCherry) (*myl7*, a cardiomyocyte gene) transgenic zebrafish embryos and found that the number of cardiomyocytes were significantly reduced (Fig. 2A) without affecting proliferation (Supplementary Fig. 3A). Considering the reduction of cardiomyocytes and close relationship between programmed cell death and cardiac injury [19–23], we used the main programmed cell death inhibitors and observed that only ferroptosis inhibitor Ferrostatin-1 (Fer-1) showed cardioprotective effects upon BHPF exposure (Fig. 2B). Fluorescence activated cell sorting (FACS) of cardiomyocytes from Tg(*myl7:* dsRed) zebrafish also showed significant reduction of the proportion of cardiomyocytes and upregulation of canonical ferroptosis biomarkers *ptgs2a* and *ptgs2b* upon BHPF exposure (Fig. 2C and Supplementary Fig. 3B). Moreover, zebrafish chronically exposed to a low concentration (0.1 μM) of BHPF resulted in the accumulation of lipid peroxides in cardiomyocytes (Supplementary Fig. 3C) and disarranged mitochondria in cardiomyocytes, with reduced cristae density (Supplementary Fig. 3D). Collectively, these findings suggest that ferroptosis participated in BHPF-induced cardiac defects.

To clarify the mechanism of ferroptosis in BHPF-induced cardiomyocyte injury, we carried out metabolomic profiling using liquid chromatography tandem mass spectrometry (LC-MS/MS) and performed pathway enrichment analysis by SMPDB (small molecule pathway database); the results indicated that the pterin biosynthesis pathway, which is one of the major ferroptosis inhibitory systems [24–28], was dysregulated in BHPF exposed zebrafish, suggesting that BHPF stimulates ferroptosis via disruption of the pterin biosynthesis pathway (Supplementary Fig. 3E and Fig. 2D). Insufficiency of tetrahydrobiopterin (BH4), a key product of the pterin biosynthesis pathway with antioxidant activity, generally leads to ferroptosis [29,30]. Since BH4 is easily oxidized, its dehydrogenated product dihydrobiopterin (BH2) is often used as a surrogate molecule for BH4 in measuring and detection. Indeed, BHPF caused a significant downregulation of BH2 levels in zebrafish (Supplementary Fig. 3F). Notably, supplementing BHPF-exposed zebrafish with exogenous BH2 reduced reactive oxygen species (ROS) accumulation (Supplementary Fig. 3G), and greatly reduced heart defects (Fig. 2E, F and Supplementary Fig. 3H). Additionally, treating the zebrafish with either Fer-1 or BH2 significantly blocked BHPF-induced expression of ferroptosis biomarkers, *ptgs2a* and *ptgs2b*, and heart failure markers, *nppa* and *nppb* (Supplementary Fig. 3I). Collectively, the above results suggest downregulation of BH4/2 as a key event for BHPF-induced ferroptosis and heart failure.

GCH1 is a rate-limiting enzyme in the biosynthesis of BH4 and plays an important role in inhibition of ferroptosis [29,30]. Therefore, we investigated whether GCH1 is dysregulated upon BHPF exposure. Western blot analysis revealed that BHPF downregulated GCH1 protein levels in a concentration-dependent manner, accompanied by a gradual reduction in BH2 levels (Fig. 2G). Importantly, overexpression of GCH1 significantly rescued BHPF-mediated downregulation of BH2 (Supplementary Fig. 3J), and repressed BHPF-induced upregulation of ferroptosis and heart failure biomarkers (Supplementary Fig. 3K), which was accompanied by relieving of BHPF-induced reduction in heart rate and cardiac viability (Fig. 2H and I). Taken together, these data suggest that BHPF triggers cardiomyocyte ferroptosis by repression of the GCH1-BH4/2 antioxidant axis, leading to cardiac injury in zebrafish. Importantly, consistent with BHPF-induced effects in zebrafish, the offspring from BHPF-gavage female mice (7.3 nM BHPF in the serum of pregnant mice) showed cardiac phenotypes (Fig. 1H and Fig. 2J), downregulation of GCH1, and upregulation of ferroptosis indicators (Fig. 2K and Supplementary Fig. 4A, B).

### BHPF reduces YTHDF2-facilitated-*gch1* translation

To elucidate the mechanism by which BHPF represses the expression of GCH1 protein, we first detected the mRNA expression of *gch1* and found that BHPF did not reduce the levels of its transcripts (Supplementary Fig. 5A). On the other hand, proteasome inhibitor MG132 could not rescue BHPF-mediated GCH1 protein downregulation (Supplementary Fig. 5B and C). These results suggested that BHPF downregulated GCH1 protein levels neither through reduction of *gch1* mRNA expression nor through proteasome-mediated GCH1 protein degradation which implied that BHPF might downregulate GCH1 protein via affecting its mRNA translation.

Since m^6^A modification is a key regulator in mRNA translation, we investigated whether there was any involvement of m^6^A modification in BHPF-induced reduction of GCH1. Using sequence-based RNA adenosine methylation site predictor (SRAMP) [31], two m^6^A-site clusters were predicted on *gch1* mRNA, respectively, locating at the 5’-UTR-CDS junction and CDS-3’-UTR junction (Supplementary Fig. 5D, top panel), and m^6^A-MeRIP-qPCR results confirmed the existence of these two m^6^A-site clusters (Supplementary Fig. 5D, bottom panel). Since m^6^A modification is mainly catalyzed by the METTL3/METTL14 complex, we knocked down METTL3 by morpholinos (*mettl3*-MO) in zebrafish and found a notable reduction of m^6^A levels on *gch1* mRNA ensuing downregulation of GCH1 protein but not mRNA levels, which was accompanied by a functional cardiac phenotype (Supplementary Fig. 5E). These results suggested that METTL3-mediated m^6^A methylation plays a role in *gch1* mRNA translation, and loss of m^6^A modification led to downregulation of the protein product from translation of *gch1* mRNA, resulting in heart defects. To test this notion, we performed polysome profiling in *mettl3*-MO zebrafish and measured *gch1* mRNA by RT-qPCR on each of the collected fractions. METTL3 knockdown resulted in a translational defect as evidenced by the shift of *gch1* mRNA from heavier to lighter polysomal fractions (Fig. 2L), supporting the fact that m^6^A methylation facilitates *gch1* mRNA translation.

We next investigated whether BHPF downregulates GCH1 via m^6^A-related mechanisms. Through evaluating the effects of BHPF on the main m^6^A modifiers by proteomic analysis, we found that only the m^6^A reader YTHDF2 showed sensitivity to BHPF (Supplementary Fig. 5F). Western blot results further confirmed BHPF-induced downregulation of YTHDF2, similar to BHPF-mediated cardiac defects (Fig. 1B and Fig. 2A), in a concentration-dependent manner (Fig. 2M). Given these data and recent studies demonstrating that YTHDF2 could regulate translation of m^6^A modified RNA [12,32,33], it indicated a possibility that BHPF-mediated downregulation of YTHDF2 disrupts translation of *gch1* mRNA and leads to ferroptosis and subsequent heart failure. Supporting this, polysome profiling assay indicated that *ythdf2* knockdown repressed translation of *gch1* mRNA (Fig. 2N) without affecting its stability (Supplementary Fig. 5G). We then found that only wild-type but not m^6^A binding defective mutant YTHDF2 (*ythdf2-flag* MUT) [34] displayed strong affinity to *gch1* mRNA (Supplementary Fig. 5H and I); additionally, *mettl3* knockdown robustly reduced binding affinity of YTHDF2 to *gch1* mRNA (Supplementary Fig. 5J), suggesting that YTHDF2 recognizes and regulates *gch1* mRNA in an m^6^A-dependent manner. To further investigate the effects of m^6^A and YTHDF2 on *gch1* mRNA translation, we carried out reporter assays with wild type (*gch1-flag* WT) and m^6^A sites mutated (A to T) *gch1* mRNA (*gch1-flag* MUT). The *gch1-flag* WT displayed a much greater affinity to YTHDF2 than the *gch1-flag* MUT (Fig. 2O, top panel), and the levels of protein product from *gch1-flag* MUT were significantly lower than that from *gch1-flag* WT mRNA (Fig. 2O, lines 1–3). As the levels of *gch1-flag* WT and MUT mRNA were similar, this supported the importance of YTHDF2 binding and m^6^A modification for *gch1* mRNA translation. In addition, YTHDF2 depletion equalized the levels of protein products of *gch1-flag* WT and MUT mRNA (Fig. 2O, lines 4–6), further verifying the role of YTHDF2 in regulation of *gch1* mRNA. Taken together, these results indicate that the translation of *gch1* is positively regulated by YTHDF2 in an m^6^A-dependent manner.

Notably, *ythdf2* knockdown in zebrafish (*ythdf2*-MO) significantly downregulated GCH1 protein without affecting its mRNA levels and induced similar cardiac injury phenotypes (Supplementary Fig. 6A) to those in BHPF-exposed and METTL3-depleted zebrafish (Fig. 1C and Supplementary Fig. 5E). Moreover, these *ythdf2* knockdown-induced functional cardiac phenotypes could be rescued by the ferroptosis inhibitor Fer-1 and BH2 (Supplementary Fig. 6B, C), suggesting that ferroptosis is a key downstream event induced by downregulation of YTHDF2-facilitated-*gch1* translation for heart failure. Remarkably, BHPF-induced cardiac defects were rescued by YTHDF2 overexpression, and *ythdf2* knockdown induced cardiac defects were relieved by GCH1 overexpression (Supplementary Fig. 6D and E), confirming the YTHDF2-GCH1-ferroptosis regulatory axis upon BHPF exposure. Moreover, these mechanistic findings were further supported by chronic BHPF exposure models and mutant zebrafish, as induction of cardiac phenotypes, downregulation of GCH1, and upregulation of biological indicators were similarly observed in chronic low-concentration BHPF exposure (0.01 μM exposure, 8.6 nM in zebrafish trunk) (Fig. 1G and Supplementary Fig. 6F) and *ythdf2* homozygous mutant (*ythdf2^−^^/^^−^*) zebrafish (Fig. 2P, Q and Supplementary Fig. 6G). Collectively, these data indicate that BHPF exposure downregulates YTHDF2, which in turn inhibits the translation of *gch1* mRNA in an m^6^A-dependent manner, thereby causing ferroptosis and leading to cardiac defects (Fig. 2R).

In addition, reduction of YTHDF2 was also observed in BHPF-exposed mice (Fig. 2K and Supplementary Fig. 7A). Both BHPF-exposed mice (Fig. 1H; Fig. 2J, K and Supplementary Fig.4 A, B) and cardiomyocyte-specific YTHDF2 conditional knockout mice (Supplementary Fig. 7B–F and Supplementary Fig. 4B, C) showed downregulation of GCH1, upregulation of ferroptosis indicators, and phenotypes similar to those observed in BHPF-exposed and YTHDF2-depleted zebrafish, reflecting evolutionary conservation of this BHPF-YTHDF2–induced circuitry. Most importantly, our preclinical trials show that sapropterin dihydrochloride (an orally active synthetic form of BH4, the major component of the clinically used drug, KUVAN^®^) can rescue heart failure in cardiomyocyte-specific *Ythdf2* conditional knockout mice (Supplementary Fig. 7G and H), suggesting a feasible therapeutic strategy for dealing with BHPF-induced cardiac defects.

### BHPF causes TNFα-driven CVP apoptosis

We then investigated the mechanism by which BHPF induced the CVP defects. Since BHPF-induced downregulation of YTHDF2 is the key event for induction of ferroptosis-dependent heart failure, we examined whether BHPF-mediated CVP defects are also driven by the same pathway. As shown in Supplementary Fig. 8A, *ythdf2* knockdown also led to CVP defects, suggesting that the CVP phenotype may correlate with ferroptosis and heart failure. Unexpectedly, ferroptosis inhibitor Fer-1 treatment failed to rescue the BHPF-induced CVP phenotype (Supplementary Fig. 8B). Therefore, BHPF-stimulated CVP defects are also induced via a YTHDF2-mediated mechanism, but independent of ferroptosis.

**Figure 3.**
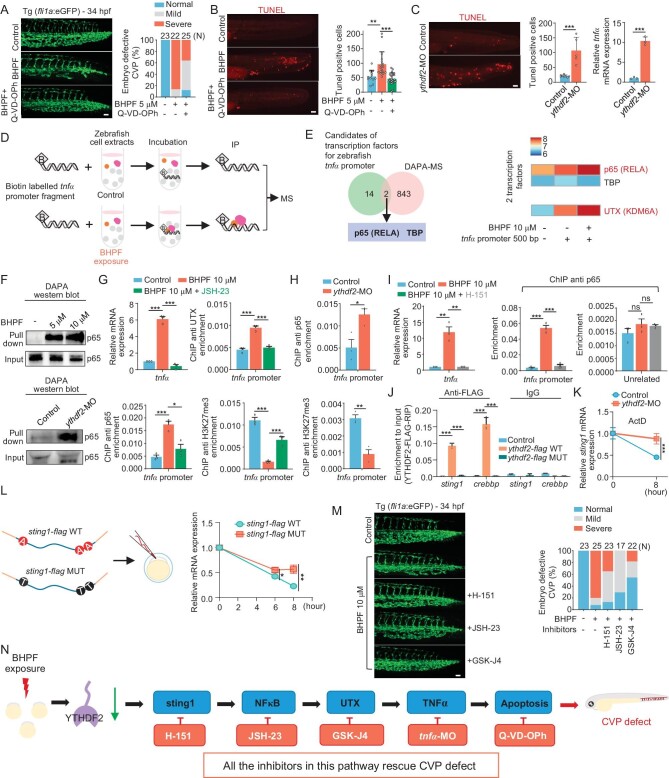
BHPF-mediated downregulation of YTHDF2 induces apoptosis via activation of STING1-NFκB/UTX-TNFα axis. A and B, Effects of apoptosis inhibitor Q-VD-OPh on BHPF-induced CVP defects (A) and apoptosis (B) in 34 hpf zebrafish embryos with quantification (*N* denotes number of embryos for each experimental group). C, Effects of YTHDF2 depletion (*ythdf2*-MO) on apoptosis (images with quantification) and *tnfα* mRNA levels in zebrafish embryos. D, Schematic illustration of the unbiased DNA oligo affinity protein assay (DAPA)-mass spectrometry method. E, Venn diagram showing overlap of the DAPA-MS identified zebrafish *tnfα* promoter-bound proteins with the predicted zebrafish *tnfα* promoter transcription factors expressed in CVP region. The heat map depicting the abundance of proteins binding to *tnfα* promoter fragment with or without BHPF exposure (right panel). The data is log_10_ transformed. F, Western blot results showing the binding affinity of p65 to *tnfα* promoter fragment upon BHPF exposure (up panel) or depletion of YTHDF2 (down panel). G, ChIP-qPCR and RT-qPCR analysis showing association of p65, UTX, and H3K27me3 levels on *tnfα* promoter and *tnfα* mRNA expression in 34 hpf embryos under BHPF exposure with or without JSH-23 treatment. H, ChIP-qPCR analysis showing the levels of *tnfα* promoter-bound p65 and H3K27me3 in 34 hpf wild type zebrafish embryos and *ythdf2* morphants. I, ChIP-qPCR and RT-qPCR analysis showing association of p65 on *tnfα* promoter and *tnfα* mRNA expression in 34 hpf embryos under BHPF exposure with or without H-151 treatment. J, Effects of m^6^A binding ability of YTHDF2 on YTHDF2 and *sting1* mRNA interaction in embryos expressing wild type (*ythdf2*-*flag* WT) or m^6^A binding defective mutant (*ythdf2*-*flag* MUT); *crebbp* was used as a positive control. K, Effects of YTHDF2 on *sting1* mRNA stability. Wild-type embryos, *ythdf2* morphants with or without 8 hours of Actinomycin D (ActD) treatment were harvested for RT-qPCR analysis. L, Line chart showing the levels of microinjected *sting1* mRNA (wild type and m^6^A modification sites mutant) in embryos at indicated time points. M, Effects of H-151, JSH-23, and GSK-J4 on BHPF-induced CVP defects with quantification (*N* denotes number of embryos for each experimental group). N, Schematic illustration of the YTHDF2-STING1-NFκB/UTX-TNFα apoptosis axis upon BHPF exposure. Suppression of any single step within this pathway (H-151 for STING, JSH-23 for NFκB, GSK-J4 for UTX, *tnfα* morpholino for TNFα, and Q-VD-OPh for apoptosis) rescued BHPF-induced CVP defects. Scale bar, 20 μm (A–C, M). Molecular biology experiment materials are zebrafish whole embryos (D–L). Zebrafish embryos were treated with BHPF from 4 hpf to the indicated time. Data are mean ± s.d. Student's t test, ns represents *P* > 0.05, * represents *P* < 0.05, ** represents *P* < 0.01, *** represents *P* < 0.001.

To identify the mechanism by which BHPF induced the CVP phenotype, we performed RNA-sequencing (RNA-seq) analysis on the wild type zebrafish after treatment with BHPF. A total of 1030 differentially expressed genes, including 423 upregulated genes and 607 downregulated genes, were identified and subjected to KEGG (Kyoto Encyclopedia of Genes and Genomes) pathway enrichment analysis. The enriched pathways among differentially expressed genes included ‘Apoptosis’ and ‘Necroptosis’, suggesting that these two programmed cell death pathways may contribute to BHPF-induced CVP defects (Supplementary Fig. 8C). Although necroptosis inhibitor Necrostatin-1 (Nec-1) could not repress BHPF-induced CVP defects (Supplementary Fig. 8B), apoptosis inhibitor Q-VD-OPh significantly decreased the BHPF-induced CVP phenotype (Fig. 3A). Additionally, BHPF-induced TUNEL signals in the CVP region could be suppressed by Q-VD-OPh, further supporting BHPF-induced TUNEL signals representing apoptotic cells (Fig. 3B). Annexin V and propidium iodide (PI) assay by flow cytometry confirmed BHPF-induced apoptosis (Supplementary Fig. 8D). Notably, 6 out of 15 differentially expressed genes enriched in the apoptosis pathway were associated with the *tnfα* signaling cascade (Supplementary Fig. 8E, left panel), and similar to the BHPF-mediated CVP defects, BHPF induced *tnfα* expression in a concentration-dependent manner (Supplementary Fig. 8E, right panel), suggesting that BHPF induced CVP defects via TNFα-dependent apoptosis. Indeed, injection of *tnfα* mRNA was sufficient to induce apoptosis around the tail region of larvae leading to CVP defects (Supplementary Fig. 8F, G) and indicating that BHPF-induced *tnfα* could induce apoptosis for CVP defects. Importantly, knockdown of TNFα (*tnfα*-MO) largely reduced BHPF-induced apoptosis and rescued the CVP defects (Supplementary Fig. 8H, I). Altogether, these findings demonstrate that TNFα-driven apoptosis, which is independent of ferroptosis, plays an important role in BHPF-induced CVP defects.

Since BHPF reduced YTHDF2 levels (Fig. 2K), and *ythdf2* knockdown led to apparent CVP defects (Supplementary Fig. 8A), we examined if TNFα-driven apoptosis was regulated by YTHDF2. Conspicuously, knockdown of *ythdf2* robustly upregulated the levels of *tnfα* transcripts and induced apoptosis in the CVP region (Fig. 3C). Collectively, these results suggest that upon BHPF exposure, downregulation of YTHDF2 plays a dual role in inducing ferroptosis-mediated cardiac injury via repression of GCH1 translation, as well as stimulating apoptosis-mediated CVP defects through enhancement of TNFα expression (Supplementary Fig. 8J).

### BHPF reduces YTHDF2 for NFκB-TNFα activation

We next investigated how downregulation of YTHDF2 enhanced TNFα expression upon BHPF exposure. By performing reporter assays, we found that both BHPF exposure and knockdown of *ythdf2* upregulated the activity of zebrafish *tnfα* promoter (Supplementary Fig. 9A). Given these data and that showing knockdown of *ythdf2* increasing *tnfα* mRNA levels (Fig. 3C), it seems likely that BHPF upregulates TNFα expression at the transcriptional level via decreasing YTHDF2 levels.

To further explore the transcriptional regulation of *tnfα* promoter by YTHDF2, we used PROMO and AnimalTFDB3 to predict potential transcription factors (TFs) on zebrafish *tnfα* promoter and found 24 potential candidates (Supplementary Fig. 9B, left panel). Based on The Zebrafish Information Network (ZFIN) database, the expression regions of 16 among these 24 transcription factors overlap spatiotemporally with CVP (Supplementary Fig. 9B, right panel). We then examined the expression levels of these 16 candidates, but none of their mRNA levels showed a clear change upon *ythdf2* knockdown (Supplementary Fig. 9C). We next carried out an unbiased DNA oligo affinity protein assay (DAPA)-mass spectrum analysis to screen for potential transcription factors bound to zebrafish *tnfα* promoter in response to YTHDF2 downregulation; two (p65 (RELA, a component of NFκB) and TBP) of the abovementioned 16 candidates were detected on 500 bp biotin labeled double strand promoter fragment of *tnfα*, and only p65 showed enhanced binding affinity to the *tnfα* promoter fragment upon BHPF treatment (Fig. 3D, E). Consistently, in the DAPA-western blot analysis, both BHPF exposure and *ythdf2* knockdown enhanced the recruitment of p65 to *tnfα* promoter (Fig. 3F). Furthermore, chromatin immunoprecipitation (ChIP) experiments showed that BHPF exposure robustly stimulated p65 binding to *tnfα* promoter accompanied by enhancement of *tnfα* expression, while repression of p65 binding to *tnfα* promoter by NFκB inhibitor, 4-Methyl-N1-(3-phenyl-propyl)-benzene-1,2-diamine (JSH-23), suppressed BHPF-induced *tnfα* expression (Fig. 3G). Collectively, BHPF-mediated downregulation of YTHDF2 stimulated p65 binding to *tnfα* promoter, which is a key step for TNFα upregulation.

Interestingly, in the DAPA-mass spectrum analysis, we noticed that similar to p65, UTX (KDM6A), a demethylase for di- and tri-methylation of lysine 27 on histone H3 (H3K27me2/3), displayed increased binding to the *tnfα* promoter upon BHPF exposure (Fig. 3E, right panel). This prompted us to ask whether UTX plays a role in NFκB-stimulated *tnfα* promoter activation upon BHPF exposure. BHPF-induced demethylation of histone H3K27 di- and tri- but not mono-methylation (Supplementary Fig. 9D), and the immunoprecipitation (IP) results showed p65 associates with UTX *in vivo* (Supplementary Fig. 9E), suggesting the possibility that p65 recruits UTX to reduce histone H3K27me2/3 and activate the *tnfα* promoter. Indeed, JSH-23 suppressed NFκB binding to the *tnfα* promoter leading to reduction of UTX recruitment and H3K27me3 demethylation (Fig. 3G). Moreover, GSK-J4, an inhibitor of UTX/JMJD, repressed histone H3K27me3 demethylation and dramatically suppressed BHPF-induced *tnfα* expression (Supplementary Fig. 9F), supporting a critical role of histone H3K27 demethylase UTX in *tnfα* promoter activation. Importantly, in agreement with the results showing a crucial role of YTHDF2 downregulation in enhancing p65 binding to the *tnfα* promoter fragment (Fig. 3F) and *tnfα* expression (Fig. 3C), *ythdf2* knockdown increased p65 binding to, and significantly reduced H3K27me3 levels in, the *tnfα* promoter region of embryos (Fig. 3H). Taken together, these results demonstrate that recruitment of p65/UTX to the *tnfα* promoter is crucial for TNFα-driven apoptosis which is induced by BHPF-mediated downregulation of YTHDF2 (Supplementary Fig. 9G).

### Reduction of YTHDF2 stabilizes *sting1* for apoptosis

Subsequently, we examined the mechanism by which downregulation of YTHDF2 triggers NFκB transcriptional activity. Rather than enhancing the mRNA levels of NFκB (Supplementary Fig. 9C), downregulation of YTHDF2 stimulated p65 binding to *tnfα* promoter (Fig. 3F and H), which is consistent with the conventional understanding of NFκB activation by the upstream kinase cascades to enhance p65 complex binding to the target gene promoter [35,36]. Thus, we screened key factors of NFκB upstream signaling pathways. RNA-seq indicated that *ythdf2* knockdown specifically upregulated *sting1* mRNA, which was verified by RT-qPCR and was also observed in BHPF-exposed zebrafish (Supplementary Fig. 9H and I). Moreover, STING1 inhibitor, H-151, suppressed BHPF-induced p65 binding to the *tnfα* promoter and blocked the induction of *tnfα* mRNA expression (Fig. 3I), indicating STING1 as a critical effector in YTHDF2-mediated regulation of the NFκB-TNFα pathway.

YTHDF2 is an m^6^A reader and exerts its functions mainly through recognizing m^6^A modified RNA, suggesting that YTHDF2 regulates *sting1* mRNA via an m^6^A-dependent manner. Applying the sequence-based RNA adenosine methylation site predictor (SRAMP) [31], three potential m^6^A sites were predicted in the CDS region of *sting1* mRNA (Supplementary Fig. 9J). m^6^A-MeRIP-qPCR as well as m^6^A-MeRIP-seq confirmed the existence of m^6^A modifications on *sting1* mRNA (Supplementary Fig. 9K, L). Considering that YTHDF2-facilitated *gch1* mRNA translation in an m^6^A-dependent manner to regulate ferroptosis (Fig. 2), we investigated whether YTHDF2 also regulated *sting1* mRNA translation. However, depletion of YTHDF2 did not repress *sting1* mRNA translation (Supplementary Fig. 9M). On the other hand, *ythdf2* knockdown directly enhanced *sting1* mRNA levels (Supplementary Fig. 9I), and both BHPF exposure and *ythdf2* knockdown upregulated m^6^A modified *sting1* mRNA (Supplementary Fig. 9L, N, O). Taken together, these data indicated that YTHDF2 negatively regulates m^6^A modified and total levels of *sting1* mRNA.

One of the major functions of YTHDF2 as an m^6^A reader is to promote m^6^A mRNA decay [33,37]. Thus, we interrogated whether YTHDF2 regulated the stability of m^6^A-*sting1* mRNA. Only WT but not m^6^A binding defective mutant YTHDF2 bound to the *sting1* mRNA (Fig. 3J), and knockdown of *mettl3* robustly reduced YTHDF2-bound *sting1* mRNA (Supplementary Fig. 9P), supporting that YTHDF2 bound to and regulated *sting1* mRNA through an m^6^A-dependent manner. Subsequently, downregulation of YTHDF2 increased *sting1* mRNA stability (Fig. 3K). Moreover, to further verify the role of m^6^A in YTHDF2-mediated regulation of *sting1* mRNA stability, we synthesized FLAG-labeled m^6^A sites mutant (A to T) as well as wild type *sting1* mRNA and injected these mRNAs into wild type embryos, and then observed their decay kinetics. This analysis showed that *sting1* mRNA with mutated m^6^A sites was more stable than the wild type (Fig. 3L). Together, these data suggested a role for m^6^A/YTHDF2 in negatively regulating *sting1* mRNA stability.

Collectively, our results demonstrate that BHPF induces apoptosis-mediated CVP defects via a sequential process of YTHDF2-STING1-NFκB/UTX-TNFα regulatory axis. Notably, inhibiting any single step in this pathway (H-151 for STING1, JSH-23 for NFκB, GSK-J4 for UTX, *tnfα* morpholino for TNFα, and Q-VD-OPh for apoptosis) significantly rescued BHPF-induced CVP defects in zebrafish embryos (Fig. 3A, M, N and Supplementary Fig. 8I).

### BHPF/YTHDF2 regulates tissue-specific PCDs

**Figure 4.**
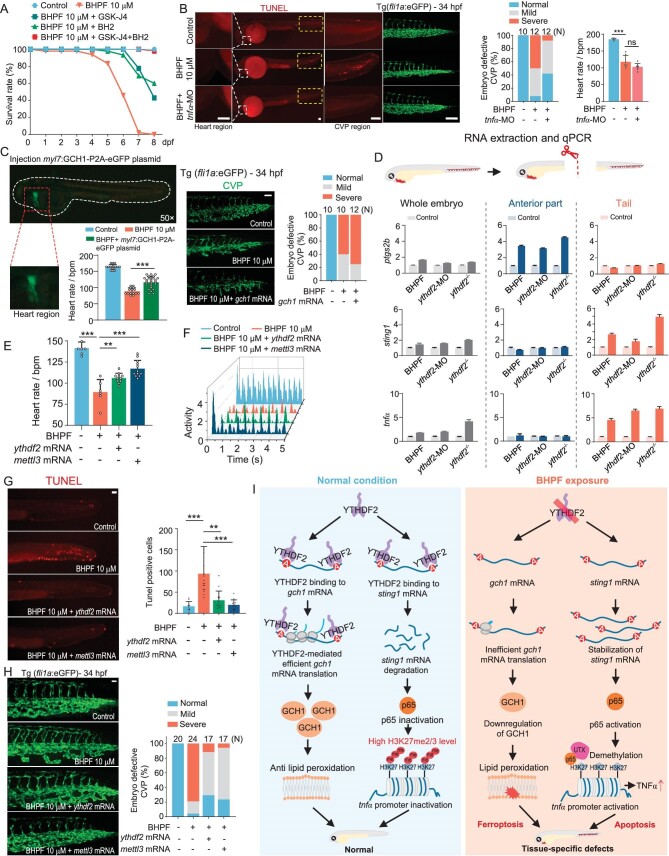
YTHDF2 acts as a bifurcation point leading to tissue-specific PCDs upon BHPF exposure. A, Survival analysis of embryos upon BHPF exposure with or without cotreatment with apoptosis and ferroptosis inhibitors; *n* = 45–50 per group. B, Effects of TNFα depletion (*tnfα*-MO) on BHPF-induced apoptosis (left panel), CVP defects (middle panel), and heart rate (right panel) in Tg(*fli1a*: eGFP) zebrafish embryos. C, Effects of cardiomyocyte-specific overexpression of *gch1* on BHPF-induced heart rate reduction (Wild type zebrafish) and CVP defects (Tg(*fli1a*: eGFP) zebrafish). Red box indicates the heart region. The white dashed line indicates the trunk region. D, Schematic diagram of whole embryo, anterior, and tail parts for gene expression analysis (top panel). Expression levels of *ptgs2a, sting1*, and *tnfα* mRNA in whole embryo, fore, and tail trunks from untreated, BHPF exposed, *ythdf2* knock-down (Morpholino) and *ythdf2* mutant zebrafish embryos (bottom panel). E and F, Rescue effects of exogenous YTHDF2 and METTL3 on BHPF-mediated reduction of heart rate (E) and heart activity (F) as analyzed by DanioScope; *n* = 6–12 per group. G and H, Rescue effects of exogenous YTHDF2 and METTL3 on BHPF-mediated CVP apoptosis (G) and CVP defects (H); *n* = 9–17 per group (G). I, Schematic diagram illustrating the mechanism of YTHDF2-mediated tissue specific programmed cell deaths (PCDs) in embryos upon BHPF exposure. *N* denotes number of embryos for each experimental group. Quantification of the images on the left is shown in the right panels. Scale bar, 100 μm (B–C, G–H). BHPF treatment of zebrafish embryos from 4 hpf to the indicated time. Data are mean ± s.d. Student's t test, ns represents *P* > 0.05, ** represents *P* < 0.01, *** represents *P* < 0.001.

We next sought to investigate whether BHPF-mediated downregulation of YTHDF2-induced ferroptosis-driven cardiac injury and apoptosis-driven CVP defects had crosstalk with each other or operated independently. Pretreatment of zebrafish embryos with ferroptosis or apoptosis inhibitor alone could partially rescue animals from lethality upon BHPF exposure, while, cotreatment of zebrafish embryos with ferroptosis and apoptosis inhibitors almost completely prevented embryonic death induced by BHPF (Fig. 4A). The additive effects of these two inhibitors suggested that ferroptosis and apoptosis are independent of each other. Next, we targeted the *tnfα* and *gch1* for BHPF/YTHDF2–induced apoptosis and ferroptosis, respectively. TUNEL staining showed BHPF-induced apoptosis only occurred at the CVP region but not at the heart region (Fig. 4B, left panel), and depletion of TNFα largely reduced BHPF-induced apoptosis and rescued the CVP defects but could not alleviate the cardiac injury (Fig. 4B, right panel). In contrast, cardiomyocyte-specific expression of GCH1 rescued cardiac defects but did not relieve CVP defects (Fig. 4C). Moreover, ferroptosis inhibitor rescued the cardiac defects (Fig. 2B and Supplementary Fig. 6B, C) but had no effect on the CVP defects (Supplementary Fig. 8B), while apoptosis inhibitor rescued CVP (Fig. 3A, B) but not the cardiac phenotypes (Fig. 2B). Collectively, the above results indicated that BHPF-induced ferroptosis-driven cardiac injury and apoptosis-driven CVP defects are two independent pathways. In addition, whole mount *in situ* hybridization (WISH) of endoderm, mesoderm and ectoderm markers revealed that BHPF treatment barely affected development of these three germ layers in zebrafish embryos (Supplementary Fig. 10A). When the BHPF exposure time of embryos was delayed from 4 hpf to 8, 12 and 24 hpf, CVP and cardiac defects were similarly observed (Supplementary Fig. 10B). Taken together, these results suggested that rather than directly affecting gastrulation, BHPF triggered ferroptosis and apoptosis to cause cardiac and CVP phenotypes in zebrafish.

To investigate how YTHDF2 displayed distinct functions in different tissues, WISH was applied to analyze the expression pattern of key factors in YTHDF2-regulated ferroptosis and apoptosis pathways. Intriguingly, zebrafish embryos robustly expressed both *ythdf2* and *tnfrsf1a* (a receptor of TNFα) mRNA at the CVP region before 34 hpf, but *gch1* mRNA expression was limited to the anterior section and showed similar expression pattern with *ythdf2* at the heart region in 48 hpf zebrafish embryos (Supplementary Fig. 10C). These different temporal and spatial localizations of *tnfrsf1a* and *gch1* might explain the tissue specificity of YTHDF2-regulated ferroptosis and apoptosis. We then found that BHPF exposure or YTHDF2 depletion (both knockdown and homozygous *ythdf2* mutant) upregulated *ptgs2b, sting1*, and *tnfα* mRNA levels in whole zebrafish extracts (Fig. 4D, left panel). Remarkably, when we separated zebrafish into anterior section and tail part, the ferroptosis marker *ptgs2b* mRNA was upregulated only in anterior sections but not in the tails, and in contrast, the upregulation of *tnfα* and *sting1* mRNAs was limited to tails upon BHPF exposure or YTHDF2 depletion (Fig. 4D, middle and right panels). The occurrence of ferroptosis and apoptosis were consistent with the co-expression patterns of *gch1/ythdf2* and *tnfrsf1a/ythdf2*, respectively (Supplementary Fig. 10C), which might explain, at least in part, YTHDF2-mediated bifurcation of different programmed cell deaths in heart and CVP regions.

Importantly, overexpression of YTHDF2 or METTL3 in BHPF-treated zebrafish embryos could partially rescue both ferroptosis-mediated heart failure (Fig. 4E, F) and apoptosis-mediated CVP defects (Fig. 4G, H), indicating m^6^A/YTHDF2-mediated regulation played a critical role in modulation of these two mechanistically independent and tissue-specific ferroptosis and apoptosis processes upon BHPF exposure in the same organism (Fig. 4I).

## DISCUSSION

BHPF at concentrations detected in pregnant people induced cardiovascular defects in zebrafish and mouse models. Notably, the fetus of participant NO. 14 likely had ventricular septal defect (VSD) based on prenatal checkup (Fig. 1A and Supplementary Fig. 12). Although this cannot be considered conclusive, it is interesting because fetus of pregnant participants with the highest levels of BHPF may develop VSD, suggesting that BHPF poses a potential threat to human fetal heart development. As humans and animals are exposed to environments polluted by BHPF [38], these findings should be made known to reduce environmental BHPF pollution and to promote further investigations on BHPF-mediated effects, including large-scale population studies. Furthermore, owing to the widespread application of BHPF-containing plastics, BHPF-releasing sources might already be inside human and animal bodies. Microplastic (MP) and nanoplastic (NP) particles, defined as being less than 5 mm and 100 nm in size, respectively, have been reported to exist widely both in the environment and the food chain [4,39]. Remarkably, recent studies have shown that microplastics can be detected in human feces and blood (with average concentration of 1.6 μg/mL in human blood) [40,41], suggesting extensive accumulation in humans. Since BHPF is released from plastic containers [42], the MP and NP particles accumulated in the blood and other organs may constantly release BHPF leading to persistent exposure to BHPF, a condition detrimental to developing fetuses.

We demonstrate that BHPF represses YTHDF2 levels to disrupt two distinct m^6^A regulatory axes and causes independent cardiovascular defects. While precisely how BHPF reduces YTHDF2 expression remains to be elucidated, it is evident that YTHDF2-regulated ferroptosis and apoptosis are two separate events in driving BHPF-induced defects in two distinct tissues. Consistently, distinct inhibitors against YTHDF2-regulated ferroptosis and apoptosis selectively rescue BHPF-induced defects in a tissue-specific manner (Figs. 2 and 3). We note that several such inhibitors, e.g. sapropterin dihydrochloride (an orally active synthetic form of BH4, the major component of the clinically used drug, KUVAN^®^) and Adalimumab (an anti-TNFα antibody) are currently available in clinic, and clinical approaches to inhibit other key regulators (e.g. STING1) are also available, suggesting multiple potential therapeutic strategies targeting BHPF-induced adverse effects [43–45]. In addition to the frequent presence in serum of pregnant people, a recent report showed that BHPF is detectable in 60% of surface waters in Beijing, China [38], further highlighting the urgency for unveiling BHPF-mediated cardiovascular defects as well as potential therapeutic countermeasures. Our preclinical trial shows that sapropterin dihydrochloride can rescue heart failure in cardiomyocyte-specific *Ythdf2* conditional knockout mice (Supplementary Fig. 7G and H), which not only further confirms the YTHDF2-GCH1-BH4 cardiac ferroptosis axis, but also suggests a feasible treatment strategy for managing the potential adverse effects caused by BHPF. Intriguingly, besides downregulation of YTHDF2 levels, we found BHPF could directly interact with YTHDF2 (K_D_ 104.9 μM) without affecting YTHDF2 protein stability (Supplementary Fig. 11). Whether BHPF binding to YTHDF2 affects the function of YTHDF2 is worth further exploration.

The main function of YTHDF2 as an m^6^A reader is m^6^A-dependent RNA decay [33]; however, YTHDF2 is also known to indirectly alter translation via modulating RNA stability (affecting the amount of non-translating mRNA to influence translation efficiency) [33] or directly bind to, and regulate translation of, particular m^6^A-modificated mRNAs [12,32]. In agreement with the m^6^A RNA degradation function of YTHDF2 [33], *ythdf2* knockdown stabilized *sting1* mRNA (Fig. 3K). On the other hand, *ythdf2* knockdown did not downregulate the translation of *sting1* mRNA (Supplementary Fig. 9M), but significantly reduced *gch1* translation (Fig. 2N) without affecting its mRNA level and stability (Supplementary Fig. 5A, G). Collectively, we found that cardiomyocyte ferroptosis is induced by downregulation of YTHDF2-facilitated translation of m^6^A-*gch1*, and CVP apoptosis is triggered by downregulation of YTHDF2-mediated m^6^A-*sting1* decay. On the other hand, the occurrence of ferroptosis and apoptosis were consistent with the spatiotemporal co-expression patterns of *gch1/ythdf2* and *tnfrsf1a/ythdf2* around the head/heart and tail regions, respectively (Fig. 4; Supplementary Fig. 10C), which also explain, in part, YTHDF2-mediated bifurcation of different programmed cell deaths in the heart and CVP regions. A combination of these results suggests that two distinct YTHDF2-mediated regulations of m^6^A as well as spatiotemporal tissue-specific co-expression patterns of ferroptosis axis genes (*gch1/ythdf2*) and apoptosis axis genes (*tnfrsf1a/ythdf2*) would play a key role for YTHDF2 in bifurcating the BHPF-induced molecular response into two distinct tissue-specific programmed cell death pathways. Altogether, these results further expand the current knowledge regarding the complex functions of m^6^A at an organismal level.

## MATERIALS AND METHODS

Detailed materials and methods are available in the Supplementary Data.

## DATA AND MATERIALS AVAILABILITY

High-throughput sequencing data that support the findings of this study have been deposited at Sequence Read Archive (SRA) under the accession number PRJNA906294 (TaxID: 7955). All the other data generated or analyzed in this study are included in the Supplementary Data.

## Supplementary Material

nwad227_Supplemental_Files
