# Supplementary material for: YTHDF2-mediated regulations bifurcate BHPF-induced programmed cell deaths
Source: Natl Sci Rev. 2023 Aug 28;10(12):nwad227. doi: 10.1093/nsr/nwad227 (PMC10751878; doi:10.1093/nsr/nwad227)
Supplement: nwad227_Supplemental_Files [file nwad227_supplemental_files.zip › SI-merge-nwad227-HSU.pdf]

YTHDF2-mediated regulations bifurcate BHPF-induced programmed cell deaths

Jiebo Lin[1,2†], Guankai Zhan[1,2†], Jinfeng Liu[1,2,3†], Yasen Maimaitiyiming[1,2,4‡], Zhiping Deng[5‡], Baohua Li[6‡], Kunhui Su[1,2], Jiafeng Chen[1,2], Siqi Sun[1,2], Wanlin Zheng[7,8], Xianghui Yu[7,8], Feng He[2,7,8], Xiaodong Cheng[9], Lingfang Wang[1,2,9], Bin Shen[10], Ziqin Yao[3,11], Xinquan Yang[3,11], Jian Zhang[1,2], Wentao He[1,2], Hengyu Wu[1,2], Hua Naranmandura[4], Kao-Jung Chang[12], Junxia Min[13], Jun Ma[2,7,8], Mikael Björklund[14,15], Peng-Fei Xu[2,7*], Fudi Wang[3,11*], Chih-Hung Hsu[1,2*]

The PDF file includes:

Supplementary Text
Methods
Supplementary Figures 1 to 12
Supplementary Tables 1 to 5

Other Supplementary Materials for this manuscript include the following:

Supplementary Movies 1 to 2
Supplementary Data 1

SUPPLEMENTARY DISCUSSION

Previous studies have indicated that YTHDF2 deficiency can trigger apoptosis in leukemia stem cells and triple-negative breast cancer cells through directly increasing TNFR2 expression and by stimulating unfolded protein accumulation leading to excessive endoplasmic reticulum stress [1, 2]. Here, we demonstrated that downregulation of YTHDF2 leads to apoptosis via m^6A-*sting1*-NFκB-TNF*α* pathway. In aggregate, these findings indicated three distinct mechanisms to induce apoptosis, but all of them were triggered by depletion of YTHDF2 for upregulation of YTHDF2-degraded mRNA, which highlights that the YTHDF2 target mRNA and YTHDF2-mediated mRNA regulation are influenced by the cellular context.

Ferroptosis, a type of programmed cell death driven by iron-dependent lipid peroxidation participating in multiple bioprocesses to maintain homeostasis, is mainly modulated by three lipid peroxide accumulation eliminating pathways: GPX4-glutathione axis, FSP1-CoQ10 axis and GCH1-BH4/2 axis [3, 4]. Although increasing evidence has demonstrated that epigenetic modifications play critical roles in ferroptosis regulation [5], the RNA modification-mediated regulation of ferroptosis remains largely unknown. Recently, it was reported that m^6A modulates GPX4-glutathione pathway by promoting NKAP induced *slc7a11* mRNA splicing and maturation, indicating involvement of m^6A modification in ferroptosis regulation [6]. In this study we revealed that YTHDF2 directly regulates ferroptosis though facilitating translation of m^6A modified *gch1* mRNA (Fig. 2). This finding not only manifested that the translation modulating function of YTHDF2 plays an important role in ferroptosis regulation, but also demonstrated that in addition to the reported m^6A-GPX4-glutathione ferroptosis axis, m^6A regulates ferroptosis also through GCH1-BH4/2 axis. On the other hand, in addition to the heart region, co-expression of *gch1/ythdf2* mRNA also occurred in the head region of zebrafish (Supplementary Fig. 10C). It was recently shown that lipid peroxidation induces ferroptosis of nerve cells in Parkinson's disease pathology, suggesting that ferroptosis plays a role in homeostasis of the nervous system [7]. Therefore, whether BHPF-mediated GCH1-BH4/2 axis ferroptosis affecting nervous system development merits further investigation.

Inhibition of CVP apoptosis rescued CVP defects (Fig. 3A, B, M and Supplementary Fig. 8H, I), indicating apoptosis around CVP region as the major reason of CVP defects upon BHPF exposure. However, as shown in Fig. 1K, BHPF-induced vascular defects only occurred at CVP region but not in other regions, suggesting that BHPF-induced CVP defects could not be simply attributed to the apoptosis of vascular endothelial cells. Consistently, previous study reported that suppressing β-catenin activity would induce apoptosis at CVP region for CVP defects, but notably, apoptotic cells were only partially overlapping with CVP endothelial cells [8], suggesting apoptosis of other types of cells involved in CVP defects. Given the complexity of cell types at the CVP region, which contain for example mural cells, hematopoietic stem and progenitor cells [9, 10], it is likely that the unique microenvironment of the CVP region is a contributing factor to CVP apoptosis upon BHPF exposure. This may partially explain why BHPF-induced vascular defects only occurred at CVP region but will need to be further investigated.

METHODS

Animal studies

All animal experiments were conducted according to the Guideline on the Humane Treatment of Laboratory Animals and approved by the Laboratory animal management and ethics committee of Zhejiang University School of Medicine (#ZJU20220391).

Mice

YTHDF2-floxed mice (*Ythdf2*$^{flox/flox}$) were obtained from our previous study[43] and *myh6*-Cre transgenic mice were purchased from the Jackson Laboratory (stock numbers 011038). CD-1 female mice were obtained from Shanghai Slac Laboratory Animal Co. LTD. Mice were kept in a controlled environment (22 ± 2 °C, 55 ± 5% humidity, 12 h light/dark cycle) under standard conditions with water and food ad libitum. Sex-matched littermates were used for experiments in order to test a single variable.

Zebrafish

Adult zebrafish strains including wild type, Tg(*fli1a*:eGFP), Tg(*gata1*:dsRed), Tg(*lcr*:eGFP), Tg(*myl7*:eGFP) were purchased from CZRC (China Zebrafish Resource Center), Tg(*myl7*:H2A-mCherry) and Tg(*myl7*:dsRed) transgenic zebrafish were gifted from Prof. Peidong Han. All zebrafish strains were raised and maintained at 28.5°C on a 14 h light/10 h dark cycle in zebrafish core facilities of Zhejiang University School of Medicine.

Generation of knockout zebrafish lines using CRISPR/Cas9

The Cas9/gRNA target site of *ythdf2* was designed and selected from the CHOPCHOP website (https://chopchop.cbu.uib.no/). gRNAs to the exon4 of the zebrafish *ythdf2* gene were synthesized as previously described[44]. The Cas9 protein (New England Biolabs, M0646T) and *ythdf2*-targeting gRNAs were co-injected into the wild-type (WT) zebrafish embryos at 1-cell stage. The *ythdf2* mutant lines were identified by analyzing the PCR product using the primer pair: Primer-F-CAGCTCCAACCATGCCTCCT, Primer-R-GGACCGGTGGATATCGTCCT.

Cardiomyocyte-specific YTHDF2 conditional knockout mice

We crossed the *Ythdf2*$^{flox/flox}$ mice with Myh6-Cre transgenic mice, yielding *Ythdf2*$^{myh6/myh6}$ (*myh6*-Cre–driven myocyte-specific YTHDF2-deletion mice) and *Ythdf2*$^{flox/flox}$ (control) offspring. The Mice were grown to 8 weeks of age for echocardiography. Then the mice were euthanized, and the hearts were isolated for histological analysis as well as gene expression analysis.

Exposure of zebrafish to Chemicals

BPA as well as its 8 substitutes (including BHPF) were dissolved in DMSO to make stock solutions of 10 mM. The specific final concentration of inhibitors/drugs in indicated assays are displayed as follows: 20 μM lysosome inhibitor CQ, 5 μM Nec-1, 10 μM VX-765, 1 μM Fer-1, 100 μM BH2, 1 μM JSH-23, 2.5 μM GSK-J4, 0.25 μM H-151, 0.5 ng Q-VD-OPh per embryo. Zebrafish embryos were subjected to indicated treatments from at 4 hpf or microinjected with indicated drug from the one cell stage unless otherwise stated. Detailed information of chemicals can be found in the Supplementary Table 1.

BHPF exposure of mice

BHPF was dissolved in 0.1% DMSO (0.1% DMSO were used as a control). CD-1 female mice were intragastrically administered with BHPF (70 μg kg^{-1} bw 2-d^{-1}) from postnatal day (PND) 24 for 1 month to pregnancy and birth to PND 7. After exposure, the offspring from BHPF-exposed mice were subjected to echocardiographic detection. Then these offspring were euthanized, and the heart were isolated for histological as well as gene expression analysis.

Morpholinos, mRNA synthesis, WISH and plasmid construct

The antisense morpholino Oligonucleotides (MOs) were purchased from Gene-Tools (https://www.genetools.com/). The *tnfα*, *ythdf2* and *mettl3* MOs used in this study were referred to previous reports [11-13]. The sequences of MOs were as follows: *tnfα* translation-blocking MO: 5'-AGCTTCATAATTGCTGTATGTCTTA-3'; *ythdf2* translation-blocking MO: 5'-TGGCTGACATTTCTCACTCCCCGGT-3'; *mettl3* translation-blocking MO: 5'-GGATGTGACTCCATGTGTCCGACAT-3'; *gch1* translation-blocking MO: 5'-CGCTCCATAATTGCCTAGACGTGTG-3'. 1 mM stock solutions were prepared in double-distilled water (ddH_2O). The mRNAs were synthesized, and the rescue experiments were performed as previously described [11]. Whole mount in situ hybridization (WISH) staining was performed with antisense digoxigenin labeled RNA probes as previously described [14]. For mRNA overexpression, the open reading frames (ORFs) of *mettl3*, *gch1*, *ythdf2* and m^6A binding defective mutant *ythdf2* were cloned from cDNA and inserted into pCS2+ vector with 3×FLAG tag. For validating m^6A related biological functions, *gch1* and *sting1* mRNAs were cloned from cDNA and inserted into pCS2+ vector with 3×FLAG tag. According to the SRAMP prediction results, all possible m^6A sites in *gch1* cDNA (62, 109, 199, 956, 964) and *sting1* cDNA (262, 1201, 1385) were mutated to T. PGL3-GFP vector inserted with *tnfα* promoter (500 bp away from TSS region) was cloned to detect *tnfα* promoter activity, PGL3-Lucifrase vector inserted with *gapdh* promoter (2000 bp away from TSS region) was cloned as a control. For RNA probe vector constructs, the probe sequences were cloned and inserted into pEASY®-Blunt Zero vector (TransGen, CB501-01). Plasmid contained *myl7* promoter were gifts from Prof. Peidong Han and *gch1* CDS were inserted in this Plasmid for cardiomyocyte specific *gch1* expression. The primers used for plasmid constructions are displayed in Supplementary Table 3.

Fluorescence-Activated Cell Sorting (FACS) by Flow Cytometry

Tg (*myl7*:dsRed) transgenic zebrafish embryos were subjected to indicated treatments before the assay. Larvae were amputated, crushed, and trypsinized. Free cells were centrifuged and washed with PBS containing 5% FBS, filtered through a 40-μm cell strainer, and counted. Sorting was conducted on a Beckman moflo Astrios EQ at excitation wavelength of 561 nm and collected in RNAiso Plus tubes (Takara, 9109) for further detection.

Annexin V-PI apoptosis assay, BrdU Cell Proliferation Assay, Reactive Oxygen Species Assay and Lipid Peroxidation Detection by Flow Cytometry

For apoptosis assay, zebrafish were digested with TrypLE™ Select (Gibco, 12563011) under shear to a single-cell suspension and the apoptotic cells were detected by using the Annexin V-FITC/PI apoptosis kit (Multi-sciences, AP101) following the manufacturers' protocols. Analysis was conducted on a CytoFLEX LX flow cytometer (Beckman) at excitation wavelength of 561 nm (PE) and 488nm (FITC).

For proliferation assay, Tg(*myl7*:dsRed) transgenic zebrafish were co-culture with 3 mg/mL BrdU (Sigma, 59-14-3) for 12 hours, then digested with TrypLE™ Select (Gibco, 12563011) under shear to a single-cell suspension. The cells were fixed in 4% paraformaldehyde and denatured by Recombinant DNase I (Takara, 2270A), followed by labeling with BrdU primary antibody (Proteintech, 66241-1-Ig) and Alexa Fluor™ 488 secondary antibody (Invitrogen, A-11001). Analysis was conducted on a CytoFLEX LX flow cytometer (Beckman) at excitation wavelength of 561 nm (PE) and 488nm (FITC).

For reactive oxygen species detection, 48 hpf zebrafish embryos were treated with liquid nitrogen, then digested with trypsin at 37°C for 15 minutes. The dissociated cells were washed twice with 1× PBS, filtered through a 40-μm cell strainer, and incubated with 10 μM DCFH-DA fluorescent probe (Beyotime, s0033s) in serum-free medium at 37°C for 20 minutes without light, washed thrice with 1×PBS. The flow cytometric data were collected on a CytoFLEX LX flow cytometer (Beckman) at excitation wavelength 488nm (FITC).

For lipid peroxidation detection, zebrafish hearts were isolated, crushed, and digested with TrypLE™ Select (Gibco, 12563011) to obtain a single-cell suspension. Then, C11-BODIPY581/591 (Invitrogen™, D3861) was added at a final concentration of 10 μM and incubated for 30 min at 37°C. Next, the supernatant was removed by centrifuge for 2 min at 1000g and the precipitated cells were washed with PBS for three times. Then, the samples were analyzed by CytoFLEX LX flow cytometer (Beckman) at an excitation wavelength of 561 nm (PE) and 488nm (FITC).

In vivo EdU Cell Proliferation for imaging

Tg(*myl7*:H2A-mCherry) transgenic zebrafish were exposed to BHPF from 4 hpf and injected with 0.5 ng EdU at 36 hpf. After 12 hours, zebrafish embryos were fixed and permeabilized as previously described [15]. The staining was conducted using the Click-iT EdU cell proliferation kit for imaging, Alexa Fluor 488 dye (Invitrogen, C10337) according to the manufacturer's instructions.

TUNEL staining

In vivo TUNEL assay of zebrafish embryos were conducted using the in-situ cell death detection kit (Roche, 12156792910) according to the manufacturer's instructions. TUNEL positive embryos were visualized under a fluorescent microscope. The number of TUNEL positive cells was counted by ImageJ.

Western blot and antibody

Cells were lysed on ice with RIPA lysis buffer (50 mM Tris-HCl; 150 mM NaCl; 5 mM EDTA; 0.1% SDS; 1% TritonX-100; 5% Glycerol) containing protease inhibitors (Biomake, B14001) to obtain whole cell lysates. After sonication, the cell lysates were centrifuged at 14000 g at 4°C for 10 minutes to obtain supernatant, or also called as whole cell lysate. Equal amounts (about 40 μg) of extracts were separated on a 4%-20% polyacrylamide gel (GenScript, M00656) and was electrically transferred onto NC membrane (GVS, 1215458) and visualized on Tanon-5200 Chemiluminescent Imaging System (Tanon Science & Technology). The antibodies used in the study are listed in Supplementary Table 2.

RNA extraction and quantitative RT-PCR

The total RNA of cells was extracted with RNAiso Plus (Takara, 9109) in line with the protocols of manufacturer. The cDNA was synthesized by PrimeScript™ RT reagent Kit with gDNA Eraser (Takara, RRO47A) according to the manufacturer's instructions. Light Cycler 480 (Roche) was used to detect the expression level of target genes using 2×Universal SYBR Green Fast qPCR Mix (ABclonal, RK21203). The examined genes were normalized to *gapdh* mRNA expression. The specific primer sequences of examined genes are listed in Supplementary Table 4.

RNA immunoprecipitation (RIP) and m^{6}A RNA immunoprecipitation (m^{6}A-MeRIP)

For RNA Immunoprecipitation (RIP), Zebrafish embryos or cells were washed twice with ice-cold PBS and lysed in 1 mL RIP lysis buffer (150 mM KCl, 25 mM Tris, 5 mM EDTA, 0.5% Triton X-100, 0.5 mM DTT, Protease inhibitor, RNase inhibitor) on ice for 30 min. Cell lysates were centrifuged at 14000 g at 4°C for 10 min. 10% volume of supernatant was collected as input, the remaining supernatant was incubated with indicated antibody and Protein A/G Magnetic Beads (Thermo Scientific, 88803) on a rotary homogenizer at 4°C for 4 h. Protein bound RNAs were immunoprecipitated with beads and the beads were washed with RIP buffer (150 mM KCl, 25 mM Tris, 5 mM EDTA, 0.5% Triton X-100) for 4 times. Then the beads were eluted with 10 μL 10% SDS, 10 μL Proteinase K and 130 μL RIP buffer at 55°C for 30 min. RNA in IP and input groups were extracted with RNAiso Plus under the protocols of manufacturer. The relative expression of RNA was detected by RT-qPCR. IP enrichment ratio was calculated as ratio of its amount in the IP to that in the input.

For m^{6}A RNA Immunoprecipitation (m^{6}A-MeRIP), zebrafish embryos were washed twice with ice-cold PBS and extracted with RNAiso Plus under the protocols of manufacturer. Extracted RNA was depleted of genomic DNA by DNase treatment (10×DNase buffer, 2 μL DNase, 1 μL RNase inhibitor) at 37°C for 30 min. The total RNA was then purified by RNAiso Plus. RNA was fragmented with Fragmentation Reagents (Invitrogen, AM8740) at 71°C for 5 min; then, Stop Solution was added immediately. 10% volume of fragmented RNA was collected as input, the remaining RNA was incubated with m^{6}A antibody (ABclonal Technology™, m^{6}A antibody #A17924) and Protein A/G Magnetic Beads on a rotary homogenizer at 4°C for 3 h. m^{6}A modified RNA fragments were immunoprecipitated with beads and the beads were washed with RIP buffer (150 mM KCl, 25 mM Tris, 5 mM EDTA, 0.5% Triton X-100) for 4 times. Then the beads were eluted with 10 μL 10% SDS, 10 μL Proteinase K and 130 μL RIP buffer at 55°C for 30 min. RNA in IP and Input group was extracted with RNAiso Plus under the protocols of manufacturer. The relative expression of RNA was detected by one-step RT-qPCR kit (TransGen, AQ211-01). IP enrichment ratio was calculated as ratio of its amount in the IP to that in the input.

Biotinylated RNA pull-down assay

Cells were washed twice with ice-cold PBS and lysed in 1 mL RIP lysis buffer on ice for 30 min. Cell lysates were centrifuged at 14000 g at 4°C for 10 min. Supernatants were subjected to BCA kit to determine protein concentration. Equal amounts (about 500 μg) of extracts were incubated with biotinylated RNA and streptavidin-beads (BioLabs, S1420) on a rotary homogenizer at 4°C for 4 h, 10% volume of supernatant (about 50 μg) was collected as input. The beads were washed with RIP buffer for 4 times. 50 μL of 2.5× loading buffer was added to each tube, and protein expression was detected by Western Blot as described above.

Biotinylated DNA pull-down assay (DAPA) and mass spectrum analysis

DAPA assays were performed as described by Hsu et al. [16], with minor modifications. Zebrafish *ythdf2* morphants or embryos treated with BHPF and DMSO (34 hpf) were collected and homogenized in 200 µL DAPA buffer (137 mM NaCl, 2.7 mM KCl, 7.7 mM NaH_2PO_4, 1.5 mM KH_2PO_4, 0.1% NP-40, 1mM EDTA, 5% glycerol, 1mM dithiothreitol)). Then, DAPA buffer was added to bring the final volume of samples to 1 mL followed by incubation on ice for 30 min. Lysates were centrifuged at 14000 g at 4°C for 10 min. Protein concentration in supernatants were determined by BCA kit. Equal amounts (about 500 µg) of extracts were incubated with 3 µg of biotinylated DNA fragments and streptavidin-beads (Roche, 11641778001) on a rotary homogenizer at 4°C overnight. The beads were washed with DAPA buffer containing 0.5% NP-40 for 4 times. 50 µL of 2.5× loading buffer was added to each tube, and changes in protein level was detected by Western Blot as described above. For mass spectrometry, samples were washed with DAPA buffer containing 0.5% NP-40 for 4 times. Next, the beads were washed twice with ice-cold 20 mM, pH 8.0 Tris-HCl with 2 mM $CaCl_2$, then liquid-free beads were resuspended in 20 mM, pH 8.0 Tris-HCl, incubated with 4 mM DTT at room temperature for 30 min, then with 6 mM iodoacetamide for 10 min. Samples were digested with trypsin (trypsin: protein ratio at 1:50) at 37°C overnight, and then formic acid was added to a final concentration of 2% to stop the reaction. Digested samples were desalted with ZipTip and subjected to LC-MS/MS analysis for protein identification.

<u>Polysome profiling</u>

Zebrafish embryos were subjected to indicated treatments before the assay. Polysome isolation from embryos and polysome profiling methods were referred to Choudhuri et al. [17] with minor modifications: polysome buffer (Tris-HCl pH 7.5, 5 mM $MgCl_2$, 140 mM NaCl) and polysome lysis buffer (polysome buffer, 0.25% NP-40, 0.5 mM DTT, 0.1mg/mL CHX, Recombinant RNase Inhibitor (40 U/mL), Protease Inhibitor Cocktail (1×). *gch1* mRNA expression was normalized to *actin* mRNA expression. The primers for polysome profiling analysis are displayed in Supplementary Table 4.

<u>Chromatin IP (ChIP)</u>

ChIP assays for zebrafish embryos were performed as described by Cunliffe et al. [18] Specifically, embryos were harvested in 50 mm petri dish and, fixed with 5 mL 1% formaldehyde for 20 minutes at room temperature by gentle shake. Subsequently, the embryos were washed once with PBS. Next, 5 mL PBS contain 0.125 M glycine was added and incubated for 5min at room temperature to quench the cross-linking process. Next, the embryos were collected in 1.5 ml tube, centrifuged to remove the supernatant, and resuspended in 1 mL of deyolking buffer (55 mM NaCl, 1.8 mM KCl, 1.25 mM $NaHCO_3$, 2.7 mM $CaCl_2$) to disrupt the embryonic yolk masses by pipette. The deyolk embryos were centrifuged for 1 min at 2000 rpm and washed with wash buffer (110 mM NaCl, 3.5 mM KCl, 2.7 mM $CaCl_2$, 10 mM Tris pH 8.5), and this process was repeated for once. Then, the embryos were homogenized in 200 µL lysis buffer (10 mM Tris pH 8.2, 10 mM EDTA, 200 mM NaCl, 0.5% NP-40, 0.5% SDS) and sonicated by ultrasonic cell crusher (90% power, 30s on /30s off, total 6 mins, Scientz08-III, Ningbo, China). Supernatants were collected and diluted 10-fold with dilution buffer (10mM Tris-HCl pH 8.0, 1.0% Triton X-100, 1mM EDTA, 0 .01% SDS, 150 mM NaCl). The soluble chromatins were incubated with the indicated antibodies listed in Supplementary Table 2 overnight in 4°C. Then, protein A/G beads were added and incubated for 2 h in 4 °C. After incubation, immunoprecipitated complexes were washed in low-salt wash buffer (0.1% SDS, 1% Triton X-100, 2 mM EDTA, 20 mM

Tris-HCl pH 8.0, 150 mM NaCl), high-salt wash buffer (0.1% SDS, 1% Triton X-100, 2 mM EDTA, 20 mM, Tris-HCl pH 8.0, 500 mM NaCl), LiCl wash buffer (0.25 M LiCl, 1% NP-40, 1% deoxycholate, 1 mM EDTA, 10 mM Tris-HCl, pH 8.0), and TE buffer (10 mM Tris pH 8.0, 1 mM EDTA), respectively for one time. Next, 150 µL elution buffer (25 mM Tris, 10 mM EDTA, 1% SDS) was added to the samples and incubated at 65°C overnight. Next day, 2 µL RNase A (10 mg/mL) was added and incubated at 65°C for 2 h. Then, 2 µL 10 mg/mL Proteinase K was added and incubated at 65°C for 2 h. Finally, the immunoprecipitated DNA fragments were extracted by a PCR purification kit (QIAGEN, 28104) and analyzed by real-time PCR (Light Cycler 480, Roche). The primers used for ChIP-qPCR are listed in Supplementary Table 4.

Protein expression and purification

The full-length YTHDF2 fused FLAG tag was expressed in BL21 (DE3) *E. coli.* until the culture reached log phase (0.3-0.6 OD_{600}). YTHDF2-FLAG protein expression was induced with 0.2 mM isopropyl β-D-1-thiogalactopyranoside (IPTG) for 16 h at 18 °C. After induction cells were collected, pelleted (at 5000 g for 5 min) and then resuspended in the following lysis buffer: 150 mM NaCl, 0.5 mM EDTA, 50 mM Tris pH 7.4-7.9, 5% glycerol, 1% TritonX-100 and supplemented with 1mM PMSF. Then, the cells were lysed by sonication (150W, 3s on/ 3s off for 2 min) and centrifuged at 12,000*g* for 20 min. Soluble protein in supernumerary was purified by glutathione agarose for 4 h at 4 °C and washed for 4 times. Finally, YTHDF2-FLAG protein was digested by GST-3C protease for 16 h at 4 °C and concentrated by concentrators (Amicon® Ultra, Germany). All protein purification steps were performed at 4 °C.

Cardiac function evaluation and histological analysis

For zebrafish embryos, videos of heart activity were recorded from 48 hpf zebrafish placed in lateral recumbency and analyzed by DanioScope (Noldus, China). Olympus FV3000 (Olympus, Japan) was used to photograph the ventricular beating process and record the ventricular end diastolic diameter. For adult zebrafish and mice, echocardiography was performed using the Vevo1100® Imaging System and Vevo Imaging Station (VisualSonics) equipped with high frequency transducers. For histological analysis, adult zebrafish and mice were euthanized and hearts were isolated, fixed in 4% paraformaldehyde overnight at 4°C. After gradient dehydration with ethanol, the samples were dealt with Xylene (Merck) and then embedded in paraffin. Then, serially sectioned at 5-µm thickness. The sections were then stained with hematoxylin and eosin (H&E) for routine histological examination using a light microscope. Sirius red staining was used to measure collagen deposits. Cardiomyocyte cross-sectional area was determined by staining with rhodamine-conjugated WGA (catalog #L3892; Sigma-Aldrich).

CVP defects evaluation

For zebrafish embryos, loops in CVP area represent network space formed by normal growth extension of initial vessels. Total vessel area quantification in the area of the caudal venous plexus and loop numbers were calculated as previously described [19].

Transmission electron microscopy

Samples of myocardium (1 mm ×2 mm ×2 mm) were quickly removed from the ventricle and immediately fixed in 3% phosphate-glutaraldehyde, post-fixed, embedded, cut, and mounted at The Electron Microscopy Core Facility of Zhejiang University.

The samples were then viewed using a Tecnai 10 (100 kV) transmission electron microscope.

Immunohistochemistry (IHC) and immunofluorescent (IF)

Serial sections were deparaffinized and hydrated in a series of gradient alcohol solution. Antigen retrieval was performed in Citrate Antigen Retrieval solution using heat-induced protocol. Then sections were incubated with corresponding primary antibody overnight. For immunohistochemistry, specific secondary antibodies were incubated and detected with the DAB IHC staining system. For immunofluorescence, fluorescent labeled secondary antibodies were used and visualized by confocal microscopy. For quantification, three adjacent sections were quantified using ImageJ software (National Institutes of Health). The antibodies used in the study are listed in Supplementary Table 2.

RNA-sequencing, m^6A-seq proteomics and metabolomics analysis

RNA-seq and m^6A-seq was performed using whole zebrafish embryos following indicated treatments. RNA-seq and m^6A-seq service were supported by LC-Bio, Hangzhou, China. Proteomics and metabolomics analysis were performed using whole zebrafish embryos (48 hpf) with or without BHPF exposure and was supported by BIOTREE TECH, Shanghai, China.

Concentration measurement of BPA substitutes in serum

The pregnant participants' serum was obtained after appropriate ethical approval from the Medical Ethics Committee of the Women's Hospital of Zhejiang University (IRB-20220116-R). The serum samples were stored in glass tubes at -80°C until analysis. We also harvested the serum from BHPF-exposed pregnant mice. Four volumes of HPLC-grade methanol were added, samples were mixed for 3 min then centrifuged at 4000 rpm for 30 min. Supernatant solutions from each sample were transferred and removed for LC-MS/MS analysis. All analytical procedures were checked for precision, reproducibility, blank contamination and linearity. Quality control was maintained by analyzing a method blank (rat serum). Detailed parameters for analyzing the targeted compounds by LC-MS/MS are listed in Supplementary Table 5.

Measurement of fluorene-9-bisphenol (BHPF) and 7,8-Dihydro-L-biopterin (BH2) in zebrafish

Zebrafish embryos with BHPF exposure were washed with culture water three times to eliminate residual BHPF. Then, the trunks of embryos were transferred in 300 μL of pure water added with 300 μL of HPLC-grade acetonitrile, the samples were homogenized for 3 min by sonication (90% power, 30s on/30s off). HPLC-grade water (300 μL) was added to each sample and after mixing, then centrifuged at 4000 rpm for 30 min. Supernatants from each sample were transferred for LC-MS/MS analysis [20]. For BH2 detection, zebrafish embryos with different treatment were collected and subjected to the same pretreatment described above, the samples after pretreatment were transferred for LC-MS/MS analysis. Detailed parameters for analyzing the targeted compounds by LC-MS/MS are listed in Supplementary Table 5.

Statistical analysis

Statistical analysis was carried out using unpaired t test followed by GraphPad Prism version 9.3.1 for Windows (GraphPad Software, San Diego, California USA,

www.graphpad.com.), and a probability value of less than 0.05 (*, $P < 0.05$) was accepted as a significant difference; **, $P < 0.01$; ***, $P < 0.001$.

Supplementary Figure 1

A

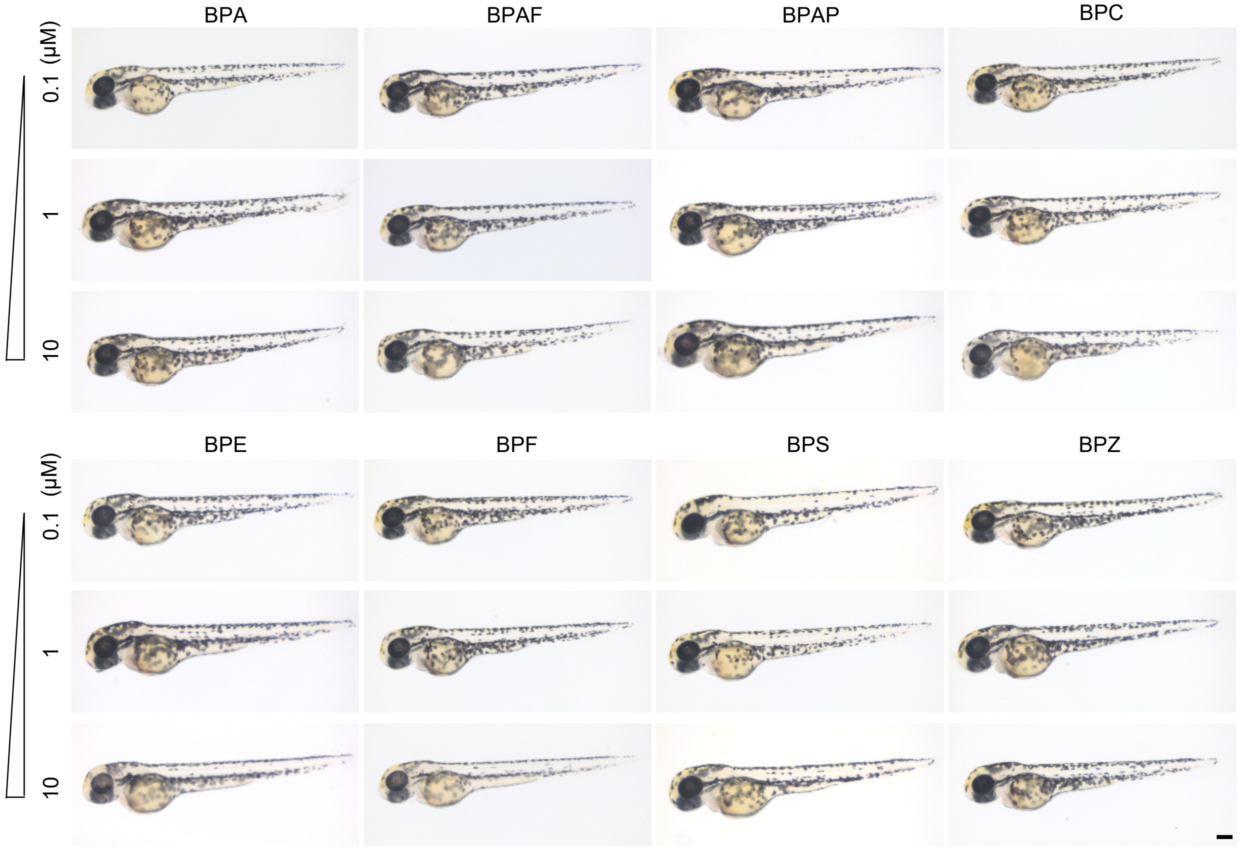

B

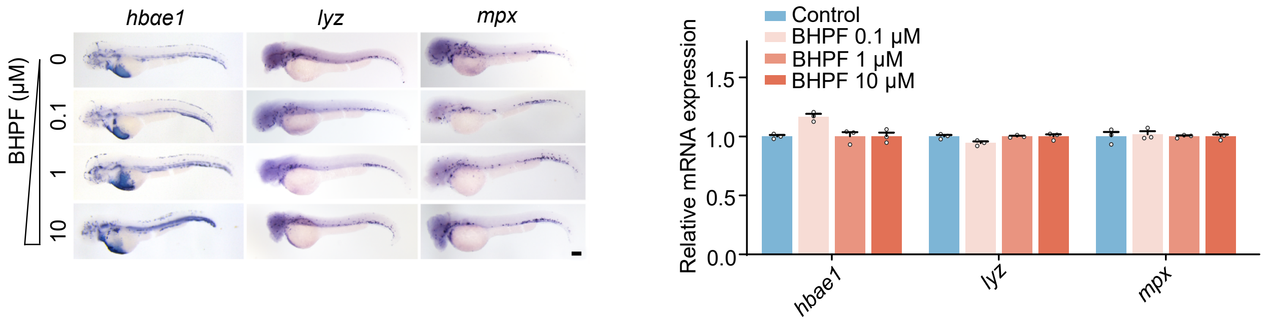

C

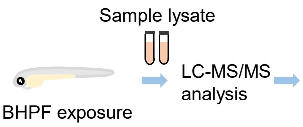

BHPF (μM) exposure*	Trunk BHPF concentrations (nM)
0.1	46.1
1	135.2
10	426.8

* Trunk concentrations in 24 hpf zebrafish larvae upon different BHPF exposure. Larval trunk volume was estimated to be 0.5 μL per embryo.

D

BHPF (μM) exposure*	BHPF concentrations (nM)
Control	ND
0.01	8.6
0.1	29.4

* Concentrations in adult zebrafish tissue upon different BHPF exposure.

Supplementary Figure 1. Morphological changes of zebrafish embryos upon exposure to BPA and its substitutes. **A**, Morphological changes of zebrafish (48 hpf) with concentration gradient exposure (0.1-10 μM) to BPA and its substitutes. **B**, Effects of BHPF exposure (0.1-10 μM) on expression of hematopoietic markers (*hbαe1* represents erythrocytes, *lyz* and *mpx* represent neutrophils). mRNA expression profiles were detected by whole mount in situ hybridization (WISH) and mRNA expression levels were measured by RT-qPCR. **C**, Measurement of BHPF concentrations in zebrafish trunk after concentration gradient (0.1-10 μM) exposure. **D**, BHPF concentrations in BHPF-exposed (0.01-0.1μM for 3 months) zebrafish, measured by LC-MS/MS analysis. Zebrafish embryos were exposed to BHPF from 4 hpf to the indicated time. Scale bar, 300 μm (**A**).

Supplementary Figure 2

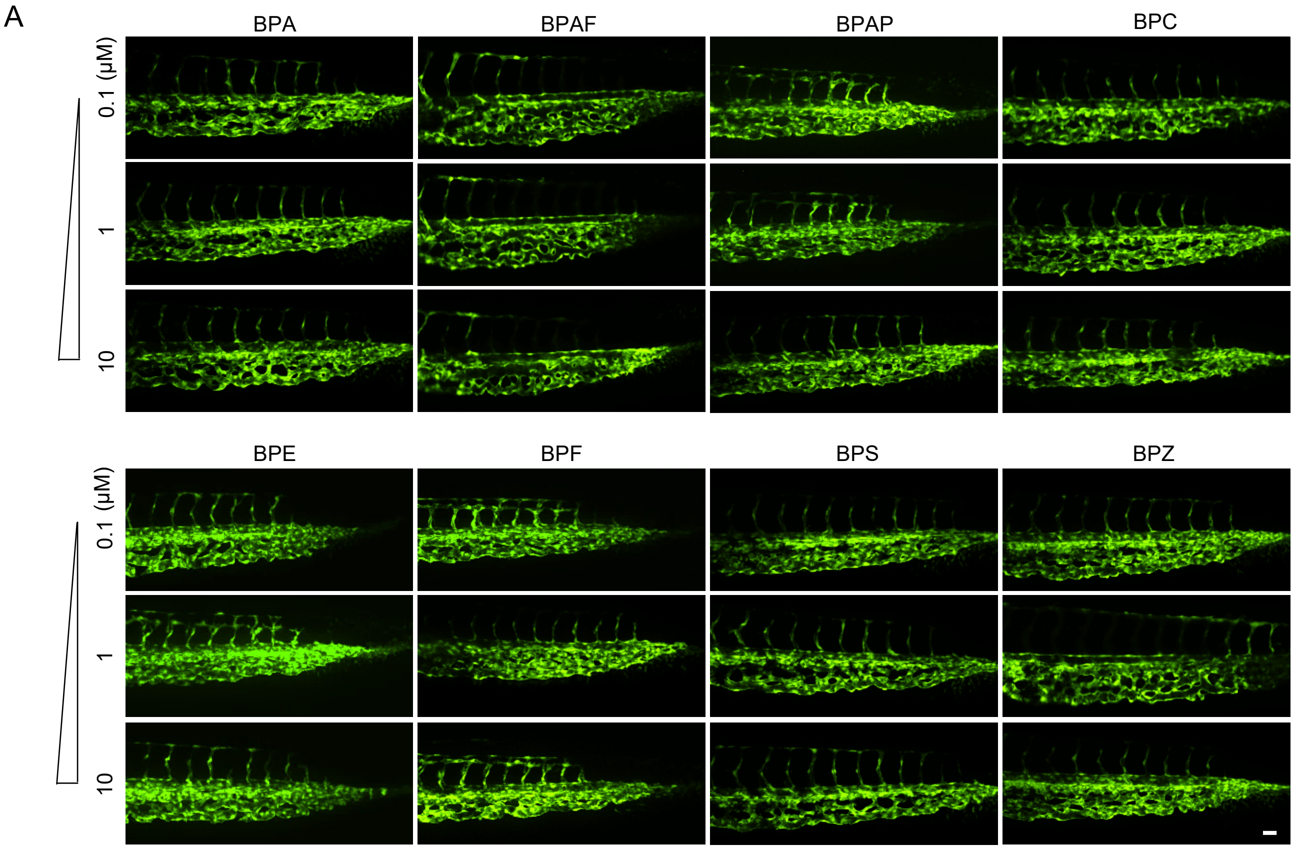

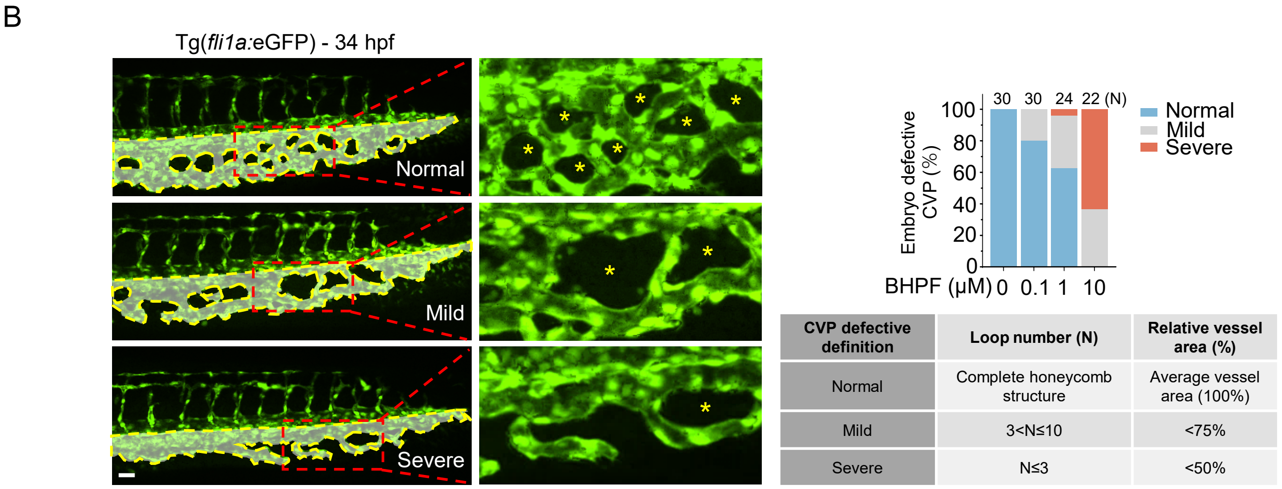

CVP defective definition	Loop number (N)	Relative vessel area (%)
Normal	Complete honeycomb structure	Average vessel area (100%)
Mild	3<N≤10	<75%
Severe	N≤3	<50%

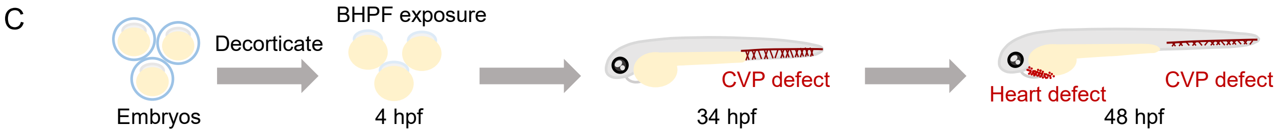

Supplementary Figure 2. Effects of BPA and its substitutes on CVP of zebrafish embryos. **A**, Confocal observation of CVP in Tg(*fli1a*:eGFP) zebrafish (34 hpf) with concentration gradient exposure (0.1-10 μM) to BPA and its substitutes. **B**, Confocal images showing BHPF-concentration-dependent CVP defects (right histogram) with definition of degree of CVP defect (right table). Yellow dashed area represents the CVP region, and the red dashed boxes indicate the magnified area, and the yellow asterisks indicate loops. **C**, Schematic diagram of BHPF-mediated defects during embryonic development in zebrafish. Percentages represent the proportion of defective phenotypes. Scale bar, 20 μm (**A**, **B**). Zebrafish embryos were exposed to BHPF from 4 hpf to the indicated time.

Supplementary Figure 3

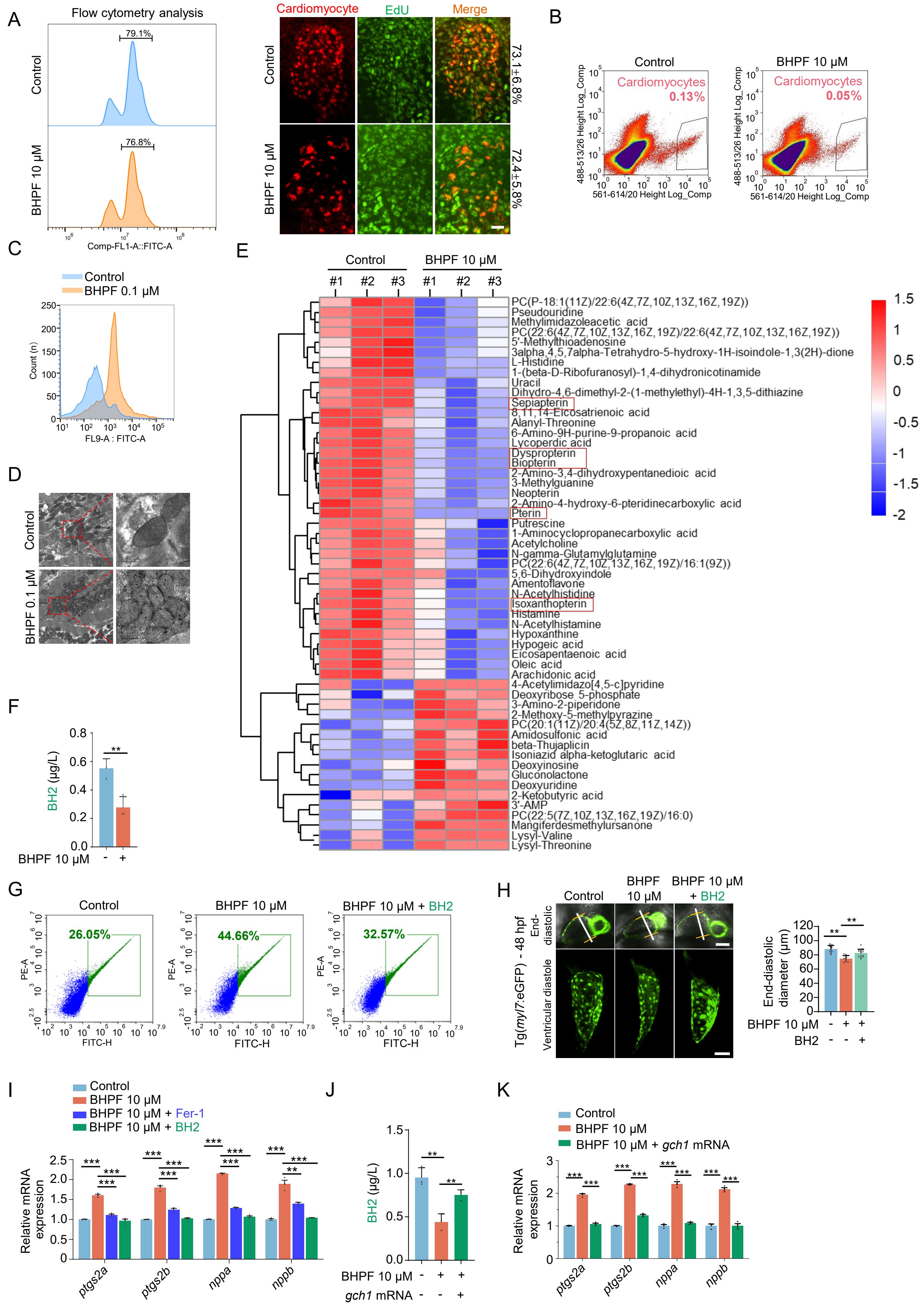

Supplementary Figure 3. BHPF represses GCH1-BH4/2 regulatory axis to induce ferroptosis. **A**, Effects of BHPF on proliferation of cardiomyocytes in zebrafish as analyzed by flow cytometry (left panel) and immunofluorescence (right panel). The percentage of EdU positive cardiomyocytes (orange)/total cardiomyocytes (red) is indicated on the right. **B**, Effects of BHPF on proportion of zebrafish cardiomyocytes in total embryonic cells as detected by FACS. **C**, Lipid peroxides levels in heart tissues isolated from control and BHPF exposed (0.1 μM for 3 months) zebrafish as measured by flow cytometry using C11-BODIPY. **D**, Electron micrographs showing mitochondria in heart tissue obtained from BHPF-exposure (0.1 μM for 3 months) zebrafish. **E**, Heatmap of BHPF-mediated differentially expressed metabolites. The red boxes indicate pterine metabolites. The data in the heatmap is log^{10} transformed. **F**, Effects of BHPF on BH2 concentration in zebrafish as detected by LC-MS/MS. **G**, Effects of exogenous BH2 supplementation in reactive oxygen species (ROS) levels in BHPF-exposed zebrafish. **H**, Effects of exogenous BH2 on end-diastolic diameter of ventricle upon BHPF exposure with quantification. The dynamic observation of ventricular end diastolic function is shown in Supplementary Video 1. n=9-15 per group. **I**, Rescue effects of exogenous BH2 and Fer-1 on *ptgs2a*, *ptgs2b*, *nppa*, and *nppb* mRNA levels in BHPF-exposed zebrafish. **J**, Rescue effects of exogenous *gch1* mRNA on BH2 concentration. **K**, Rescue effects of exogenous *gch1* mRNA on *ptgs2a*, *ptgs2b*, *nppa* and *nppb* mRNA levels in BHPF-exposed zebrafish. Molecular biology experiment materials are zebrafish whole embryos (**A**-**B**, **E**-**K**). All zebrafish embryos were treated with BHPF from 4 hpf to 48 hpf (**A**-**B**, **E**-**K**) or 3 mpf (**C**-**D**). Scale bar, 50 μm (**A**, **H**). Data are mean±s.d. Student's t test, ns represents p>0.05, * represents p<0.05, ** represents p<0.01, *** represents p<0.001.

Supplementary Figure 4

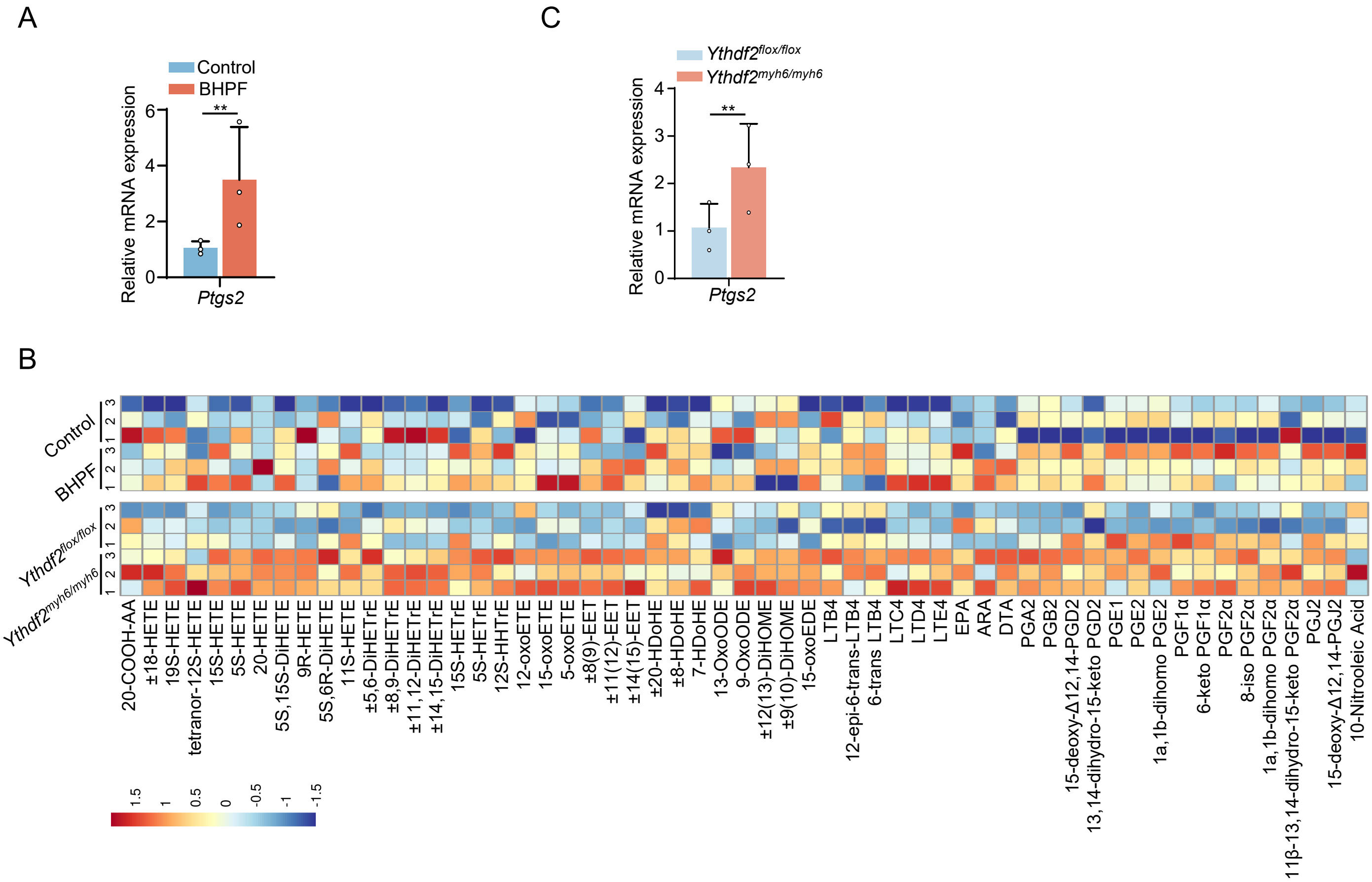

Supplementary Figure 4. Changes of lipid peroxides in the heart of offspring from BHPF-gavage female mice and Ythdf2 conditional knockout mice. A, Detection of *ptgs2* mRNA level by RT-qPCR in the offspring of the control and BHPF-exposed mice. **B**, A heatmap showing relative quantities of oxidized lipid metabolites in the control and BHPF-gavage mice as well as wild type ($Ythdf2^{flox/flox}$) and $Ythdf2^{myh6/myh6}$ mice by LC-MS/MS. n=3 per group. All molecular biology experiment materials are hearts of mice. **C**, Detection of *ptgs2* mRNA expression level by RT-qPCR in wild type ($Ythdf2^{flox/flox}$) and $Ythdf2^{myh6/myh6}$ mice.

Supplementary Figure 5

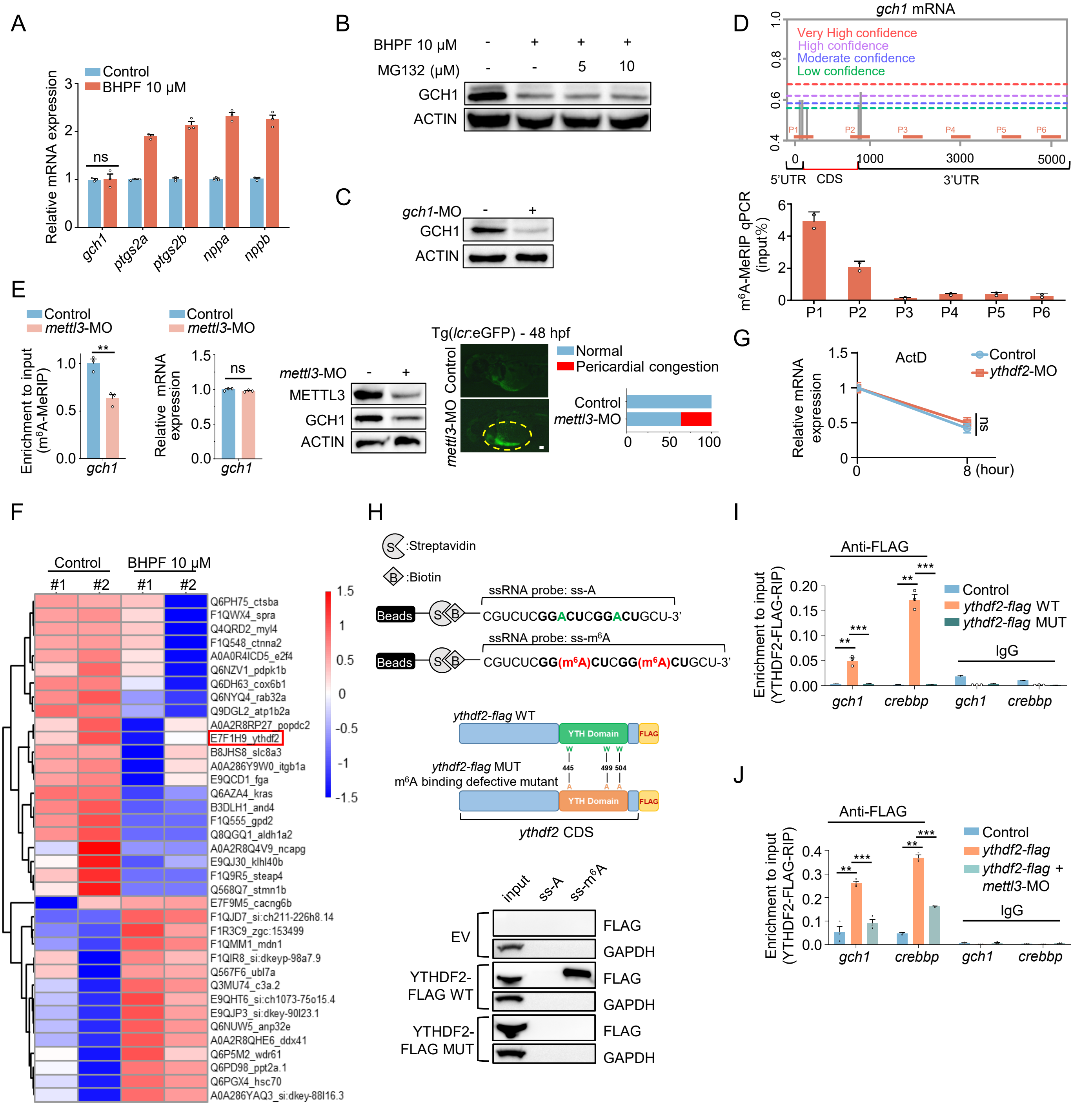

Supplementary Figure 5. BHPF downregulates m^{6}A-dependent YTHDF2-mediated GCH1 translation. **A**, Effects of BHPF exposure on *gch1*, *ptgs2a*, *ptgs2b*, *nppa* and *nppb* mRNA levels. **B**, Effects of MG132 on BHPF-mediated reduction of GCH1 protein. **C**, Validation of GCH1 antibody by *gch1* morphants. **D**, Prediction of m^{6}A modification sites on *gch1* mRNA by SRAMP database (top panel), and validation of m^{6}A modification on *gch1* mRNA by m^{6}A-MeRIP-qPCR (bottom panel). P1-P6 indicate the regions of PCR amplicons. **E**, Detection of m^{6}A modification on *gch1* mRNA by m^{6}A-MeRIP-qPCR (left panel), measurement of *gch1* mRNA and protein levels (middle panel), and fluorescence microscopy observation as well as quantification of pericardial congestion in wild type zebrafish and *mettl3* morphants. The yellow dashed circle indicates pericardial congestion (right panel). **F**, Proteomic heatmap of BHPF-mediated differentially expressed proteins (The data in the heatmap is $\log_{10}$ transformed). The red box indicates YTHDF2. **G**, Effects of YTHDF2 on *gch1* mRNA stability. Wild-type embryos, *ythdf2* morphants with or without 8 hours of Actinomycin D (ActD) treatment were harvested for RT-qPCR analysis. **H**, Schematic representation of single-stranded RNA probes with methylated (red) or unmethylated (green) adenosine (left panel). Schematic diagram shows the mutant sites on m^{6}A binding defective mutant YTHDF2 (right panel). The m^{6}A binding ability of wild type and mutant YTHDF2 was verified by RNA oligo pulldown-western blotting analysis (bottom panel). **I**, Effects of m^{6}A binding ability of YTHDF2 on YTHDF2 and *gch1* mRNA interaction in embryos expressing wild type (*ythdf2-flag* WT) or m^{6}A binding defective mutant (*ythdf2-flag* MUT). *crebbp* was used as a positive control. **J**, Effects of m^{6}A modification on YTHDF2 and *gch1* mRNA interaction in wild type and *mettl3* morphants (*mettl3*-MO). All molecular biology experiment materials are zebrafish whole embryos. All zebrafish embryos were exposed to BHPF from 4 hpf and collected at 48 hpf (**A**-**F**) or 10 hpf (**H**-**J**). Data are mean±s.d. Student's t test, ns represents $p>0.05$, * represents $p<0.05$, ** represents $p<0.01$, *** represents $p<0.001$.

Supplementary Figure 6

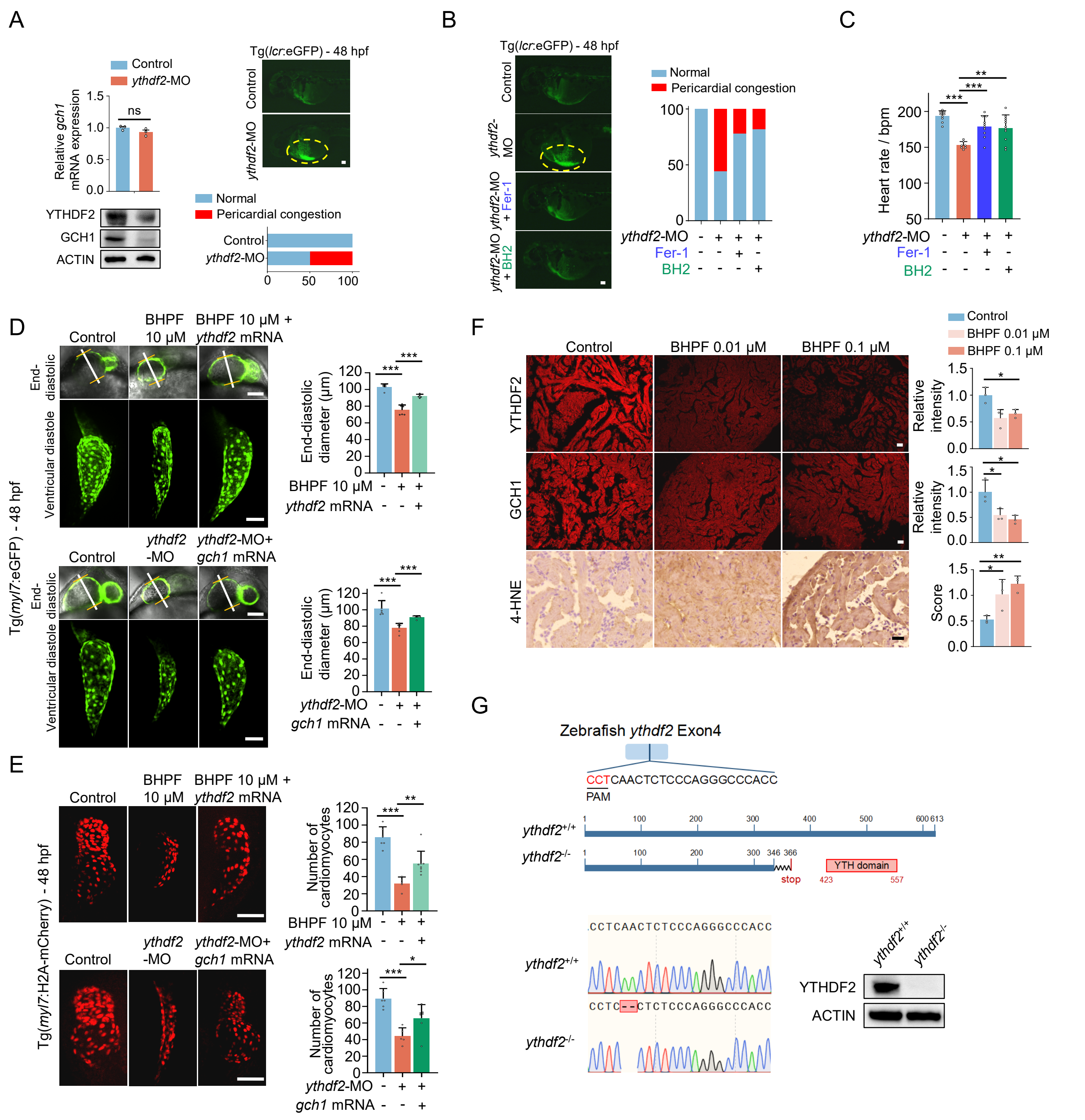

Supplementary Figure 6. Ythdf2 morphants and *ythdf2* mutant (*ythdf2*$^{-/-}$) zebrafish exhibit cardiac defects and GCH1 downregulation. **A**, Detection of *gch1* mRNA and protein levels, as well as fluorescence microscopy observation of pericardial congestion in wild type and *ythdf2* knockdown (*ythdf2*-MO) embryos with quantification. The yellow dashed circle indicates pericardial congestion. n=10 per group. **B**, Effects of exogenous BH2 and Fer-1 on pericardial congestion in *ythdf2* morphants (*ythdf2*-MO) with quantification. n=9-11 per group. **C**, Effects of exogenous BH2 and Fer-1 on heart rate (bpm refers to beats per minute) in *ythdf2* morphants. n=7-11 per group. **D** and **E**, Effects of exogenous *ythdf2* or *gch1* mRNA on end-diastolic diameter of ventricle (**D**) and the number of zebrafish cardiomyocytes (**E**) upon BHPF exposure or in *ythdf2* morphants (*ythdf2*-MO) with quantification. The dynamic observation of ventricular end diastolic function is shown in Supplementary Video 2. n=5-8 per group. **F,** Images of immunofluorescence for YTHDF2, GCH1 staining and immunohistochemistry for 4-HNE in BHPF-exposure (0.01-0.1 μM for 3 months) zebrafish with quantification. **G**, Diagram showing CRISPR-Cas9 approach used to generate *ythdf2* mutant zebrafish, which resulted in 2 bp deletion and a premature STOP codon in exon 4. The predicted truncated protein has 366 amino acids instead of 613, lacking the entire YTH domain of the YTHDF2 protein (top panel). The sequence validation is shown in the left panel. Right panel displays YTHDF2 protein levels in wild-type (*ythdf2*$^{+/+}$) and *ythdf2* mutant (*ythdf2*$^{-/-}$) zebrafish by western blot (bottom panel). All zebrafish embryos were collected at 48 hpf (**A-E**). Scale bar, 100 μm (**A-B**), 50 μm (**D-E**) or 20 μm (**F**). Data are mean±s.d. Student's t test, ns represents $p>0.05$, * represents $p<0.05$, ** represents $p<0.01$, *** represents $p<0.001$.

Supplementary Figure 7

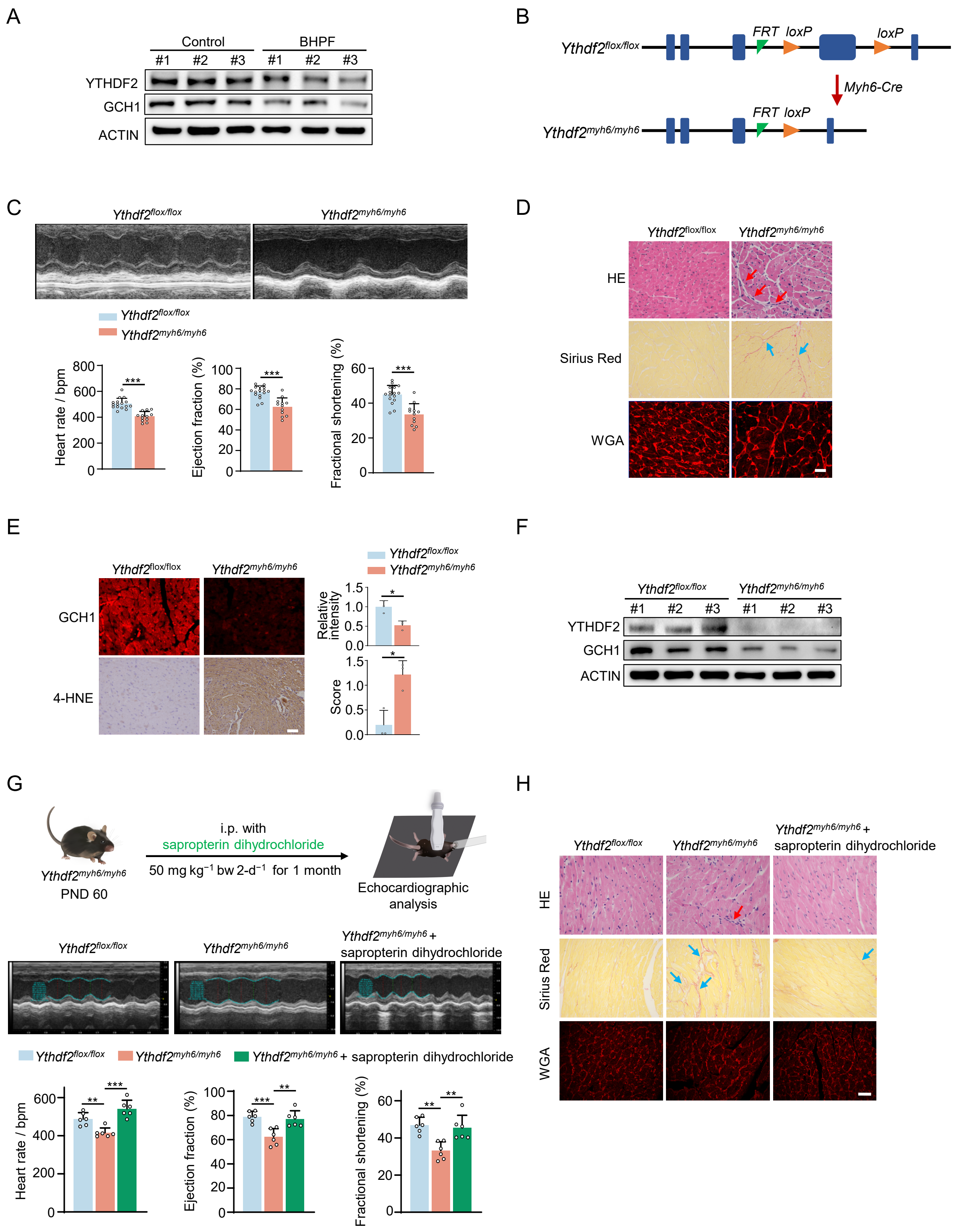

Supplementary Figure 7. BHPF-YTHDF2-GCH1-BH4 cardiac ferroptosis axis exists in mouse. **A**, Detection of YTHDF2 and GCH1 protein expression levels by western blot in the offspring of the control and BHPF-exposed mice. **B**, Schematic diagram depicting the strategy used to generate cardiomyocyte conditional knockout mouse of *ythdf2* (*Ythdf2*$^{myh6/myh6}$). **C**, Representative echocardiograms, summary of heart rate, cardiac ejection fraction (EF) and fractional shortening (FS) in wild type (*Ythdf2*$^{flox/flox}$) and *Ythdf2*$^{myh6/myh6}$ mice. n=12-16 per group. **D**, Representative images of cardiac sections stained with Hematoxylin and eosin (HE), Sirius red or Wheat germ agglutinin (WGA) in hearts obtained from wild type (*Ythdf2*$^{flox/flox}$) and *Ythdf2*$^{myh6/myh6}$ mice. **E**, Immunofluorescence for GCH1 staining and immunohistochemistry for 4-HNE in wild type (*Ythdf2*$^{flox/flox}$) and *Ythdf2*$^{myh6/myh6}$ mice with quantification. **F,** Detection of YTHDF2 and GCH1 protein expression levels in hearts by western blot in wild type (*Ythdf2*$^{flox/flox}$) and *Ythdf2*$^{myh6/myh6}$ mice. **G**, Representative echocardiograms, summary of heart rate, cardiac ejection fraction (EF) and fractional shortening (FS) in wild type (*Ythdf2*$^{flox/flox}$), *Ythdf2*$^{myh6/myh6}$ mice and *Ythdf2*$^{myh6/myh6}$ mice with sapropterin dihydrochloride supplementation. n=12-16 per group. **H**, Representative images of cardiac sections stained with Hematoxylin and eosin (HE), Sirius red or Wheat germ agglutinin (WGA) in hearts obtained from wild type (*Ythdf2*$^{flox/flox}$), *Ythdf2*$^{myh6/myh6}$ mice, and *Ythdf2*$^{myh6/myh6}$ mice with sapropterin dihydrochloride supplementation. Molecular biology experiment materials are hearts of mice (**A** and **F**). Scale bar, 20 μm (**D**-**E** and **H**). Data are mean±s.d. Student's t test, * represents $p<0.05$, **represents $p<0.01$, *** represents $p<0.001$.

Supplementary Figure 8

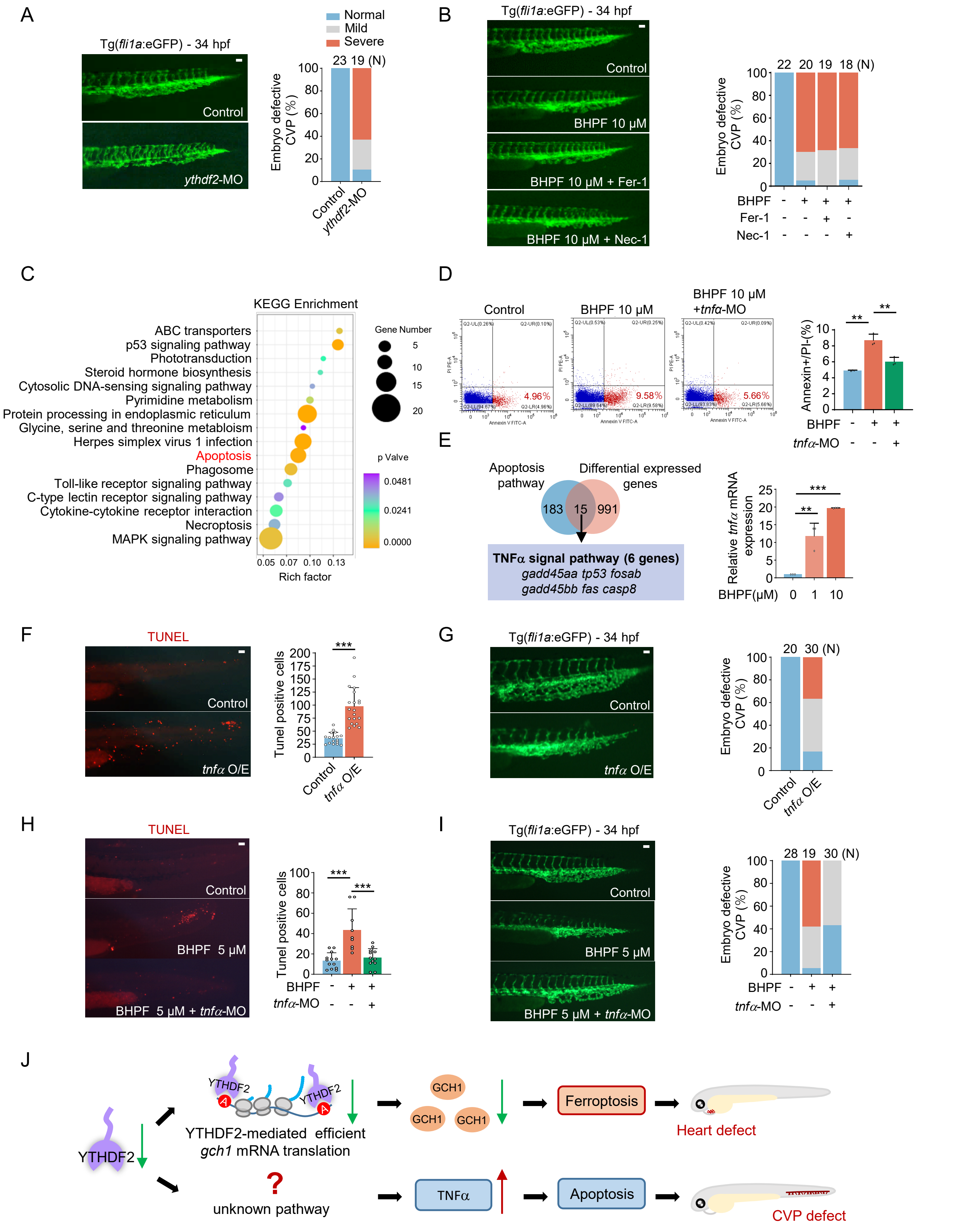

Supplementary Figure 8. BHPF-mediated YTHDF2 reduction stimulates TNFα-dependent CPV apoptosis. A, Images of the CVP in wild type embryos and *ythdf2* morphants at 34 hpf with quantification (N denotes number of embryos for each experimental group). **B**, Images of the CVP in BHPF exposed embryos with or without treatment of Fer-1 or Nec-1 with quantification (N denotes number of embryos for each experimental group). **C**, KEGG pathway enrichment map of BHPF-induced differentially expressed genes in 48 hpf zebrafish embryos. **D**, Effects of BHPF exposure on apoptosis detected by flow cytometry using Annexin V/Propidium iodide staining in zebrafish. **E**, Venn diagram showing the overlap of apoptosis signal pathway related genes and differentially expressed genes upon BHPF exposure with a list of TNFα signaling cascade related genes among overlapped genes (left panel). Relative expression levels of *tnfα* mRNA in embryos with concentration gradient exposure (1-10 μM) to BHPF (right panel). **F** and **G**, Images of apoptotic cells at the tail region (**F**) and images of CVP (**G**) in untreated and *tnfα* mRNA microinjected Tg(*fli1a*:eGFP) zebrafish embryos (34 hpf) with quantification. n=17-20 per group (**F**). N denotes number of embryos for each experimental group. O/E refers to over-expression. **H** and **I**, Effects of TNFα depletion (*tnfα*-MO) on BHPF-induced apoptosis (**H**) and CVP defects (**I**) in Tg(*fli1a*:eGFP) zebrafish embryos (34 hpf) with quantification. n=9-13 per group (**H**). N denotes number of embryos for each experimental group. **J**, Schematic diagram showing YTHDF2 downregulation induced apoptosis leading to CVP defects by upregulated *tnfα* levels, which was independent of ferroptosis. Molecular biology experiment materials are zebrafish whole embryos (**C**-**E**). Zebrafish embryos were exposed to BHPF from 4 hpf to the indicated time. Scale bar, 20 μm (**A**-**B**, **F**-**I**). Data are mean±s.d. Student's t test, **represents $p<0.01$, *** represents $p<0.001$.

Supplementary Figure 9

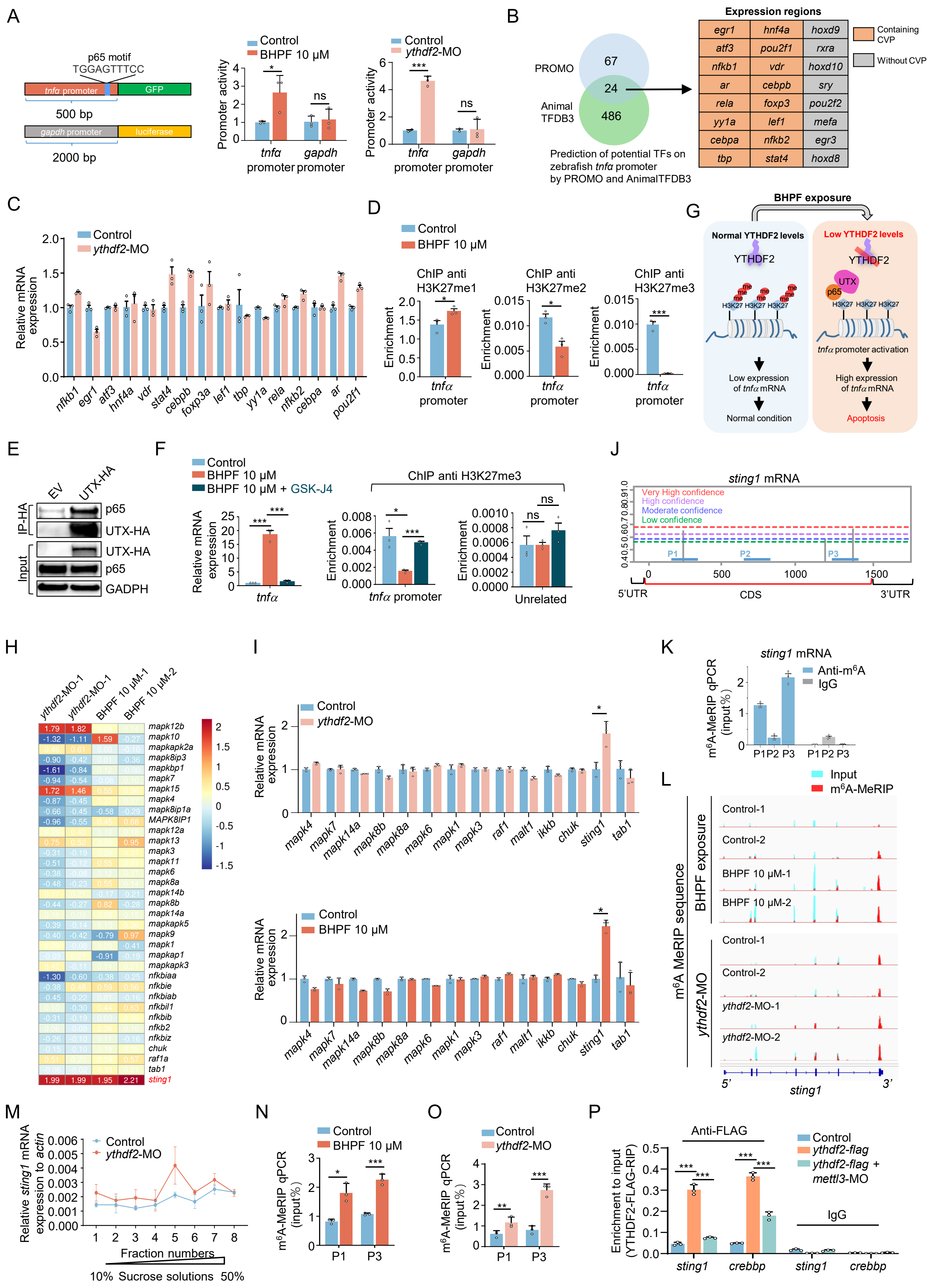

Supplementary Figure 9. Identification of BHPF-mediated YTHDF2-STING1-NFκB/UTX-TNF*α* apoptosis regulatory axis. **A**, Effects of BHPF exposure or *ythdf2* knockdown (*ythdf2*-MO) on *tnfα* promoter activity. Zebrafish embryos with BHPF exposure or *ythdf2*-MO were injected with *tnfα* promoter reporter plasmids for reporter assay. **B**, Venn diagram showing the overlap of predicted transcription factors on *tnfα* promoter via PROMO and AnimalTFDB3 database (left panel). Based on the database of The Zebrafish Information Network (ZFIN), 16 out of 24 potential transcription factors which are expressed in regions containing CVP (right panel). **C**, Effects of YTHDF2 depletion on expression of 16 candidates from (**B**). **D**, ChIP-qPCR analysis showing H3K27me1, H3K27me2 and H3K27me3 levels on *tnfα* promoter in zebrafish embryos upon BHPF-exposure. **E**, Immunoprecipitation (IP) results showing zebrafish UTX association with human p65 in 293T cell line. **F**, ChIP-qPCR and RT-qPCR analyses showing H3K27me3 levels on *tnfα* promoter and *tnfα* mRNA expression in 34 hpf embryos under BHPF exposure with or without UTX inhibitor GSK-J4 treatment. **G**, Schematic illustration showing downregulation of YTHDF2 stimulating p65/UTX binding to and activating *tnfα* promoter. **H,** A heatmap showing relative quantities of NFκB upstream signal cascade related genes in *ythdf2* knockdown (*ythdf2*-MO) or BHPF-exposed zebrafish. **I**, Effects of YTHDF2 depletion (top panel) and BHPF exposure (bottom panel) on mRNA expression of major NFκB upstream signal cascade related genes in 34 hpf embryos. **J-L**, Prediction of m^6A modification sites on *sting1* mRNA by SRAMP database (**J**), and confirmation of m^6A modification on *sting1* mRNA by m^6A-MeRIP-qPCR (**K**) as well as m^6A-MeRIP-seq (**L**). P1-P3 indicates regions of PCR amplicons. **M**, Measurement of *sting1* mRNA translation profile in wild type zebrafish and *ythdf2* morphants by polysome profiling. **N** and **O**, Detection of m^6A enrichment on *sting1* mRNA in embryos under BHPF exposure (**N**) and *ythdf2* knockdown (*ythdf2*-MO) (**O**) in 34 hpf zebrafish. The regions of PCR amplicons were indicated in (**J**). **P**, Effects of m^6A modification on YTHDF2 and *sting1* mRNA interaction in wild type zebrafish and *mettl3* morphants (*mettl3*-MO). *crebbp* was used as a positive control. Molecular biology experiment materials are zebrafish whole embryos (**A**-**D**, **F**, **H**-**I**, **K**-**P**). Zebrafish embryos were exposed to BHPF from 4 hpf to the indicated time. Data are mean±s.d. Student's t test, ns represents p>0.05, * represents p<0.05, ** represents p<0.01, *** represents p<0.001.

Supplementary Figure 10

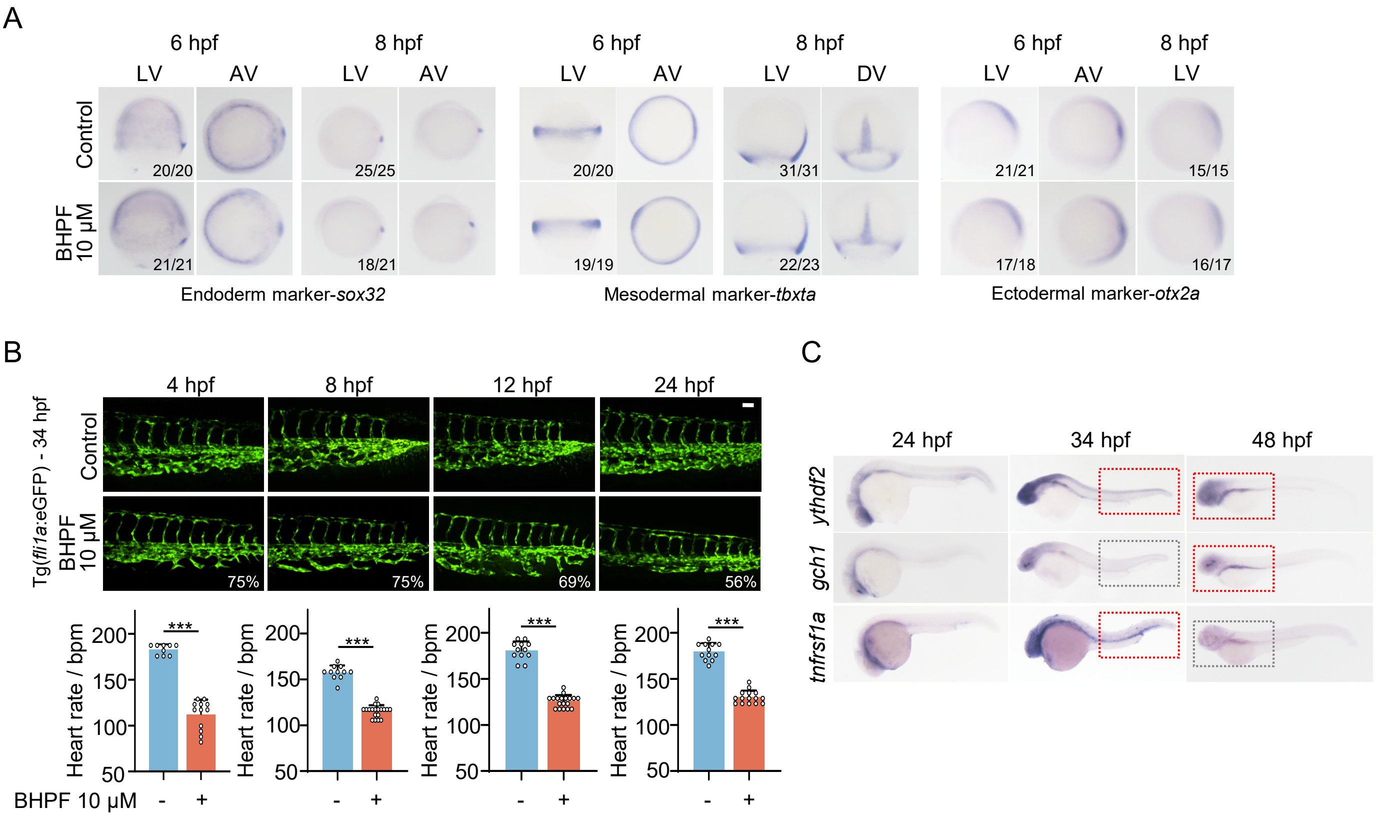

Supplementary Figure 10. BHPF barely affects development of endoderm, mesoderm and, ectoderm in zebrafish embryos. A, Effects of BHPF exposure on endoderm, mesoderm and ectoderm development in 6 and 8 hpf embryos by whole mount in situ hybridization (WISH). *sox32* was used as an endodermal marker, *tbxta* as a mesodermal marker, and *otx2a* as an ectodermal marker. LV: lateral view, AV: animal view, DV: dorsal view. **B**, Effects of BHPF exposure from different developmental stages on CVP defects (with percentages) as well as heart rate. n=9-19 per group. **C**, Gene expression pattern analysis of *ythdf2*, *gch1*, *tnfrsf1a* in 24, 34, 48 hpf embryos by whole mount in situ hybridization (WISH). Red dashed boxes indicate that there is corresponding gene transcript expression at that location, and grey dashed boxes indicate no overlapping. Scale bar, 20 μm. Zebrafish embryos were exposed to BHPF from 4 hpf to the indicated time. Data are mean ± s.d. Student's t test, *** represents p<0.001.

Supplementary Figure 11

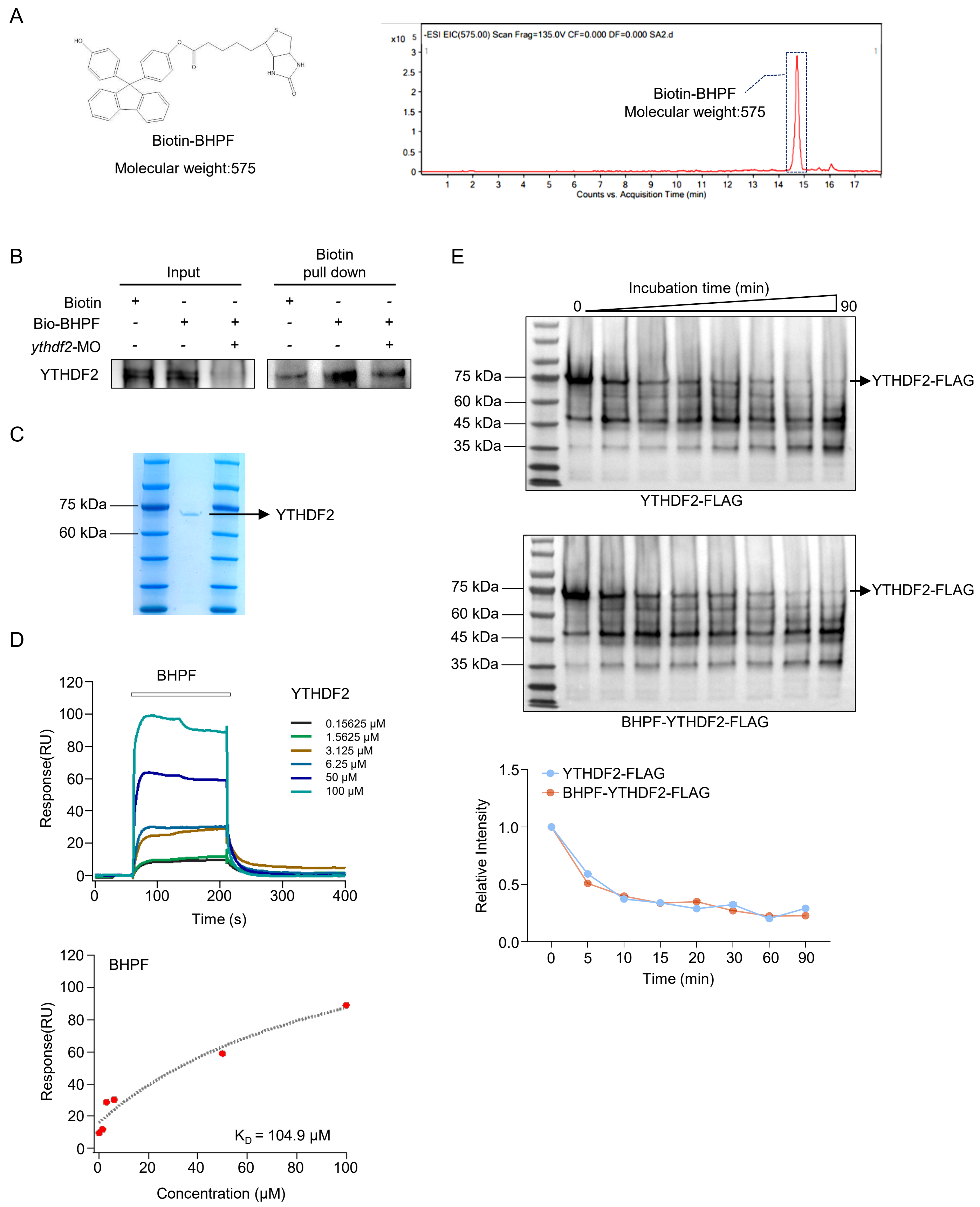

Supplementary Figure 11. BHPF binds to YTHDF2 protein without affecting its stability. **A**, Molecular Formula of Biotin-BHPF and Mass Spectrometry validation. **B**, Pull-down analysis of the binding between Biotin-BHPF and endogenous YTHDF2. **C**, Coomassie Brilliant Blue staining of purified YTHDF2 protein. **D**, SPR analysis of the binding between YTHDF2 and BHPF at different concentrations. **E**, Western Blot analysis of degradation of YTHDF2-FLAG and BHPF-YTHDF2-FLAG complex. The relative YTHDF2 signal is quantified in the bottom panel.

Supplementary Figure 12

超 声 号：[redacted]　　仪器型号：GE VolusonE10_9857　　日　期：2021-09-07 08:55

姓　　名：[redacted]　　性　　别：女　　年　龄：32岁

病 案 号：[redacted]　　病床号：　　病区：　　送检医生：[redacted]

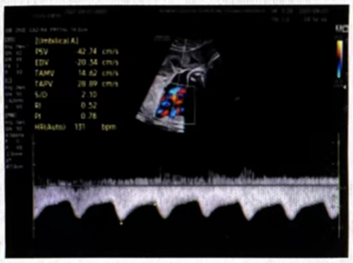

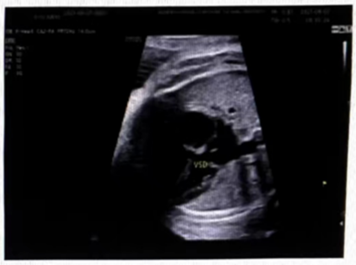

影像所见：

胎位：LOA

胎心：　131次 / 分

胎动：　可及

双顶径：9.4cm ，　头围约31.5cm

股骨长：7.0cm　，腹围30.69cm

胎盘：前壁 GrⅡ级

羊水：3-4cm

脐动脉S/D比值：　2.1　　PI:0.78

根据目前超声检查，未见明显胎儿无脑儿、严重脑膨出、严重开放性脊柱裂、胸腹壁缺损伴内脏外翻、单腔心、致命性软骨发育不全现象。

备注：胎儿心脏室间隔膜周部回声隐约见中断0.31cm。因胎位、胎龄关系，胎儿颜面部、部分肢体等结构显示不清。

检查意见：

宫内孕，单活胎

胎儿心脏室缺可能

Supplementary Figure 12. The maternity checkup of No. 14 participant. The red boxes described that echocardiogram shows a 0.31 cm interruption around the ventricular septum of the fetal heart and suggests a potential fetal VSD.

Supplementary Table 1. Reagents and inhibitors.

Reagents and inhibitors

Reagent	Source	Catalog#
BHPF	Sigma-Aldrich	399981
BPC	Sigma-Aldrich	06723
BPE	Sigma-Aldrich	04487
BPS	Sigma-Aldrich	43034
BPF	Sigma-Aldrich	51453
BPZ	Sigma-Aldrich	77923
BPAF	Sigma-Aldrich	90477
BPAP	Sigma-Aldrich	38753
BPA	Sigma-Aldrich	239658
Fer-1	MCE	HY-100579
BH2	TargetMol	T19160
H-151	MCE	HY-112693
JSH-23	MCE	HY-13982
GSK-J4	MCE	HY-15648B
VX765	MCE	HY-13205
CQ	TargetMol	T0194
Nec-1	MCE	HY-15760
Q-VD-OPh	MCE	HY-12305
Actinomycin D	MCE	HY-17559
MG132	Selleck	S2619
Cycloheximide	Cell Signaling Technology	#2112S
Recombinant RNase Inhibitor	Takara	#2313A
Recombinant DNase I	Takara	#2270A

Supplementary Table 2. Primary and secondary antibodies

Used for western blotting (WB) and Chromatin immunoprecipitation assay (ChIP).

**Antibody**	**Source, Catalog#**	**Dilutions**
H3K27me1	ABclonal, #A2361	WB 1:1000, ChIP 1:100
H3K27me2	ABclonal, #A2362	WB 1:1000, ChIP 1:100
H3K27me3	ABclonal, #A2363	WB 1:1000, ChIP 1:100
GAPDH	Proteintech, #60004-1-Ig	WB 1:10000
ACTIN	Proteintech, #20536-1-AP	WB 1:2000
GCH1	ABclonal, #A10616	WB 1:1000
METTL3	ABclonal, #A8370	WB 1:1000
YTHDF2	Proteintech, #24744-1-AP	WB 1:1000
4-HNE	Invitrogen, #MA5-27570	IHC 1:50
m^6A	Abclonal, #A17924	IP 1:200
HA	Santa Cruz, #sc-7392	WB 1:500
FLAG	Sigma Aldrich, #F1804	WB 1:500

p65 (RELA)	ABclonal, #A2547	WB 1:1000, ChIP 1:100
KDM6A (UTX)	ABclonal, #A8159	WB 1:1000, ChIP 1:100
Anti-mouse HRP	Shanghai Beyotime, #A0216	WB 1:5000
Anti-rabbit HRP	Shanghai Beyotime, #A0208	WB 1:3000

Supplementary Table 3. Primers used for plasmid construction.

Construct	Forward (5'-3')	Reverse (5'-3')
pCS2+-YTHDF2-3×FLAG	GGATCCGATTACAAAGACCACGATGGT	GAATTCGAATCGATGGGATCCTGCAA
pCS2+-YTHDF2	ATGTCAGCCAGCAGCCTTCTG	CTTTACACGTCCTTGGCGATCCTG
pCS2+-GCH1-3×FLAG	GGATCCGATTACAAAGACCACGATGGT	GAATTCGAATCGATGGGATCCTGCAA
pCS2+-GCH1	ATGGAGCGCTCCAAACAGAAACC	GCTCCGGATCAGGGTCAGGAAC
pEASY-YTHDF2	ATGTCAGCCAGCAGCCTTCTG	ATGTTTTATTGGAGGTGGTGGCAGATTTG
pEASY-GCH1	ATGGAGCGCTCCAAACAGAAACC	TCAGCTCCGGATCAGGGTCAGG
pEASY-TNFRSF1A	CTTGTACGTCGGCTTGGAGT	ACGTCCCTCAATCACATTCTCA
pEASY-SOX32	ATGTATCTCGACCGGATGCTC	TATGTATTGACATATTCATT
pEASY-TBXTA	CGCTGTCAAAGCAACAGTAT	TTTGACAGAAAAAAAAAGAAA
pEASY-OTX2A	CCGTCAGCATTGCTTATCAGC	AAACACACTTGTTCGGGAAG
MYL7-GCH1	AGTGACCCCCGGGGCCACCATGGAGCGCTCCAAACAGAAACCG	AGAAATTAGTAGCTCCGGATCCGCTCCGGATCAGGGTC
pCS2-eGFP-P2A-MYL7	GGATCCGGAGCTACTAATTTCTCCTTGCTTAAGCAAGCTGGTG	GGTGGCCCCGGGGGTCA

Supplementary Table 4. Primers used for cDNA amplification and RT-qPCR.

Name	Forward (5'-3')	Reverse (5'-3')
gch1	CCAGGGGCTCCTCAAAACTC	CCCGGCCAAAAATGGGAACT
nppa	AGGCATCAGAGAGAGCCGTA	TGCTTCGGGTCGACAATAGG
nppb	CACATGAGGACATTCCCGCT	GCTCTTCAGCTCGGGTTTCT
ptgs2a	CACACATGGCATTCGCAACA	TGCATTGAAAGATTGATAGC GCA
ptgs2b	AGATGTGGATGCTGTGGAGC	AAGCCCACTTTCCCACCAAA
tnfα	AGAGTCGGGCGTTTTGGAT	CCTGGGTCTTATGGAGCGTG
gapdh	TGAGGTTAAGGCAGAAGGCG	TGACTCTCTTTGCACCACCC
actin	CCCTGGAGAAGAGCTACGAG	TGGCATACAGGTCCTTACGG
flag-gch1	AGACCACGATGGTGACTACA	TCTGGATCTACGTAATACGA CTCAC
flag-sting1	TAATGGCGCACTGCAAGCCAC	CTTTGTAGTCACCATCGTGGT CTTTG
crebbp	GCACGGAGACAGAGGAAGAC	CGAGACTGTGACGGTGAAGA
egfp	TAAACGGCCACAAGTTCAGC	AAGTCGTGCTGCTTCATGTG
luciferase	ACTGGGACGAAGACGAACAC	TGCTCCAAAACAACAACGGC
egr1	TCCTCGTCATCCACATCCAG	AGTCAGGGATCATTGGCACA
atf3	ACAAGGAAGTTCTTCGCCAC	ATTCGACGAGTGTTTGTGCA
ar	AGTTAGGAGAGCGGCAACTT	TTGGCGTTCTTGTAGGACCT
nfκb1	GATGACATTCAGGTGCGCTT	CGGATTTCCTCTTCAGCTGC
hnf4a	CCTACACCACGCTGGAGTTT	TGGTTTTTGCGTACACTGCG
vdr	CTGTTCTTGTGCAGTGCTGT	AGAAACCCTTGCATCCCTCA
cebpb	GTCCGCAAAAGCAGAGACAA	AGTCATTAAGCACCACGGGA
stat4	CTTGGATTTCAAAAGAAAGGCA AAT	CTGGAACAGACTCTGGGCTG
foxp3a	ACCTACGCCTCCATGATACG	CCTTCCTTCAACACGCACAA
lef1	CACGAGCAGAGAAAGGAGCA	TTCCTTGCGGGCTAATTCGT
nfκb2	GAGCAAGCTGTGTGGGATTC	ATCTCTCAGCAGCCTCACTC
rela	ATCACAGGGTCCTCGATGTC	GAGGGCTCCATCTTTTGCTG
yy1a	GGAAGGCGTTTGTGGAAAGT	GCAAACGTACGGTCTGTCTC
tbp	CCGTAAACTTGGGGTGCAAA	TGCCAATCGAGACTGTTCCT
pou2f1b	AACCGCAGACAGAAGGAGAA	GAAACTGATGCCGGAGACAC
cebpa	CGGAGACTACGACTACCACC	TCCCGAGGCTCTTGTTTGAT
hbae1	CGATGCTCTCTCCAGGATGT	TCCGGCATTAAGGTCATCGA
lyz	GATACGGGGAAGGACTACGG	CAGCCCGTCCATTTTCACAA
mpx	GTTGAAACAGCCAGCTCGAA	TCACAGCCTGTCATACGTGT
tab1	AGGTTACACGAGGAGCAACA	CACTGCTGTCTGTCTCAAGC
sting1	TTCTCTGGAGTGTCGGAACC	TCTGGAACTGGGTGGACTTC
chuk	CAAGACCGGGTGCTAAGAGA	TTCTCCGCTCTGACTCACTG
raf1a	TGAAGGTTCGAGGACTCCAG	ACATCTTTGGCACCGGAAAC

malt1	CACTCTGGTCCAAAGCACAC	CTTCCCGTTTCCTCCAGAGT
mapk1	CTAAAGCGCTGGACCTGTTG	TCCAGCTCCATGTCGAACTT
mapk3	AAATGTGGAGCAAGCACTGG	CTGGTAATTGGCCTGGAAGC
mapk8b	CCACAACAAACTCAAGGCGA	TCCCATTCCAGCACTTCCTT
mapk14a	GTATCGTGCCCCTGAGATCA	TGGGCATCTGAGGAAGTGAG
mapk4	CCACATTACTCGCACAAGGG	GTCCTCCTCTCTGATGACGG
mapk6	CCTTGCCAAGTTGCTACCTG	TCCTCAATGTGGAAGGGGTG
mapk7	TCTCTCTCGTTCGCTTGGAA	CTGGACAGGAGTTGGAGGAG
ikkb	ATACCAGGAGCAGAGAGCCA	CGGCTTGAACCACCAGTTTG
mapk8a	TGTGGTCTGTCGGCTGTATCA	AAGAACTCCTGAGTTGGCGT
tnfα promoter	ACGAAAATGTCACCACCACA	TCGAGTTTGCTGGGTTGTTC
unrelate	TTTGCGCATCCTAAAACTCC	ATTGCACATGTCATCGGAGA
tnfα promoter 500 bp biotin	CAAAACAAACCATGGTTGTTCA ATGAC	GTTCAGAATAACTGCTTCTTC TTTCT
gch1-m^{6}A-*p1*	AGAGAGCGCTGAGTTTGACA	TGATCGCTCCGTCAGTTTCT
gch1-m^{6}A-*p2*	TCGAAGCGACTCACATGTGT	GTGTTTTGGCAGTGGAGGAG
gch1-m^{6}A-*p3*	ACTGGTTTTGGTAGTTCACAAGG	ATGAGGACTCTTCATAGGCA TG
gch1-m^{6}A-*p4*	TTTTGTAAGTGTATCGTCCCAGA	TGTACTAATGCACTGGACGC
gch1-m^{6}A-*p5*	ATTTCCGCTTTTTGCTGAATAC	GTACCTTATTTCTTTCCAATA TTG
gch1-m^{6}A-*p6*	TCACATCTGAGAAGAGACGGAG	TTCTCTGCATTGTCGGGTGA
sting1-m^{6}A-*p1*	GGGTTTTCTTACTCTGGCCGT	CTGCCATGATACCTTTGCCTT
sting1-m^{6}A-*p2*	GAGGTGAACGTCAGAGAATACT CT	GTGGGTGATCTTGTAGACGC
sting1-m^{6}A-*p3*	CCCTCATGTTCAGCCGACC	CCCCCAAAAAACTTTAAGAA TTGTGT
Mice-actin	GGCTGTATTCCCCTCCATCG	AGTTGGTAACAATGCCATGT
Mice-ptgs2	TTCAACACACTCTATCACTGGC	AGAAGCGTTTGCGGTACTCA T

Supplementary Table 5. MRM conditions used for LC/MS/MS analysis of analytes

Compound	Precursor （m/z）	Ion product （m/z）	Fragmentor (V)	Collision Energy (eV)
BHPF	349	256	177	28
BPAF	335	265	121	20
BPAP	289	274	111	20
BPC	279	35	106	16
BPE	213	198.1	106	16
BPF	199	93	116	24
BPS	249	108	162	28
BPZ	267	223	162	32
BH2	240	196	101	8

Supplementary Movie 1. Dynamic observation of BH2-mediated effects on end-diastolic diameter of ventricle upon BHPF exposure.
The Olympus FV3000 confocal microscope was used to dynamically detect the effects of exogenous BH2 on end-diastolic diameter of ventricle upon BHPF exposure.

Supplementary Movie 2. Dynamic observation of ventricular end diastolic function.
The Olympus FV3000 confocal microscope was used to dynamically detect the effects of exogenous *ythdf2* or *gch1* mRNA on end-diastolic diameter of ventricle upon BHPF exposure or in *ythdf2* morphants (*ythdf2*-MO).

Supplementary Data 1. (separate file)

Excel file showing **A** (sheet 1), m^6A MeRIP targets, which were defined as overlapped genes between the list of $\log_2$(IP/input)>3 genes in both *ythdf2*-MO and BHPF-exposure zebrafish. **B** (sheet 2, related to Supplementary Fig. 3E), metabolomic profiling data.

REFERENCES

1. Paris J, Morgan M, Campos J *et al.* Targeting the RNA m6A Reader YTHDF2 Selectively Compromises Cancer Stem Cells in Acute Myeloid Leukemia. *Cell Stem Cell*. 2019; **25**(1): 137-148. doi: 10.1016/j.stem.2019.03.021

2. Einstein JM, Perelis M, Chaim IA *et al.* Inhibition of YTHDF2 triggers proteotoxic cell death in MYC-driven breast cancer. *Mol Cell*. 2021; **81**(1): 3048–3064. doi: 10.1016/j.molcel.2021.06.014

3. Hadian K, Stockwell BR. SnapShot: Ferroptosis. *Cell*. 2020; **181**(5): 1188-1188.e1181. doi: 10.1016/j.cell.2020.04.039

4. Chen J, Li X, Ge C *et al.* The multifaceted role of ferroptosis in liver disease. *Cell Death Differ*. 2022; **29**(3): 467-480. doi: 10.1038/s41418-022-00941-0

5. Lee J, Ji HY, Kim MS *et al.* Epigenetic reprogramming of epithelial-mesenchymal transition promotes ferroptosis of head and neck cancer. *Redox Biol*. 2020; **37**: 101697. doi: 10.1016/j.redox.2020.101697

6. Sun S, Gao T, Pang B *et al.* RNA binding protein NKAP protects glioblastoma cells from ferroptosis by promoting SLC7A11 mRNA splicing in an m(6)A-dependent manner. *Cell Death Dis*. 2022; **13**(1): 73. doi: 10.1038/s41419-022-04524-2

7. Tian R, Abarientos A, Hong J *et al.* Genome-wide CRISPRi/a screens in human neurons link lysosomal failure to ferroptosis. *Nat Neurosci*. 2021; **24**(7): 1020-1034. doi: 10.1038/s41593-021-00862-0

8. Kashiwada T, Fukuhara S, Terai K *et al.* β-Catenin-dependent transcription is central to Bmp-mediated formation of venous vessels. *Development*. 2015; **142**(3): 497-509. doi: 10.1242/dev.115576

9. Ando K, Fukuhara S, Izumi N *et al.* Clarification of mural cell coverage of vascular endothelial cells by live imaging of zebrafish. *Development*. 2016; **143**(8): 1328-1339. doi: 10.1242/dev.132654

10. Li D, Xue W, Li M *et al.* VCAM-1+ macrophages guide the homing of HSPCs to a vascular niche. *Nature*. 2018; **564**(7734): 119-124. doi: 10.1038/s41586-018-0709-7

11. Zhang C, Chen Y, Sun B *et al.* m(6)A modulates haematopoietic stem and progenitor cell specification. *Nature*. 2017; **549**(7671): 273-276. doi: 10.1038/nature23883

12. Ping XL, Sun BF, Wang L *et al.* Mammalian WTAP is a regulatory subunit of the RNA N6-methyladenosine methyltransferase. *Cell Res*. 2014; **24**(2): 177-189. doi: 10.1038/cr.2014.3

13. Hall CJ, Sanderson LE, Lawrence LM *et al.* Blocking fatty acid-fueled mROS production within macrophages alleviates acute gouty inflammation. *J Clin Invest*. 2018; **128**(5): 1752-1771. doi: 10.1172/jci94584

14. Thisse B, Thisse C. In Situ Hybridization on Whole-Mount Zebrafish Embryos and Young Larvae. In: Nielsen BS (ed.) *In Situ Hybridization Protocols*. New York, NY: Springer New York; 2014. 53-67.

15. Neef AB, Luedtke NW. Dynamic metabolic labeling of DNA in vivo with arabinosyl nucleosides. *Proc Natl Acad Sci U S A*. 2011; **108**(51): 20404-20409. doi: 10.1073/pnas.1101126108

16. Hsu CH, Peng KL, Jhang HC *et al.* The HPV E6 oncoprotein targets histone methyltransferases for modulating specific gene transcription. *Oncogene*. 2012; **31**(18): 2335-2349. doi: 10.1038/onc.2011.415

17. Choudhuri A, Maitra U, Evans T. Translation initiation factor eIF3h targets specific transcripts to polysomes during embryogenesis. *Proc Natl Acad Sci U S A*. 2013; **110**(24): 9818-9823. doi: 10.1073/pnas.1302934110

18. Cunliffe VT. Histone modifications in zebrafish development. In: III HWD, Westerfield M, Zon LI (eds.). *Methods Cell Biol*2016. 361-385.

19. Okasato R, Kano K, Kise R *et al.* An ATX-LPA(6)-Galpha(13)-ROCK axis shapes and maintains caudal vein plexus in zebrafish. *iScience*. 2021; **24**(11): 103254. doi: 10.1016/j.isci.2021.103254

20. Moreman J, Lee O, Trznadel M *et al.* Acute Toxicity, Teratogenic, and Estrogenic Effects of Bisphenol A and Its Alternative Replacements Bisphenol S, Bisphenol F, and Bisphenol AF in Zebrafish Embryo-Larvae. *Environ Sci Technol*. 2017; **51**(21): 12796-12805. doi: 10.1021/acs.est.7b03283
